# Exploring the Plasma Proteome: Identifying Hub Proteins linking Aging, Homeostasis, and Organ Function

**DOI:** 10.7150/ijms.107750

**Published:** 2025-02-10

**Authors:** Juan Jiao, Fei Gao, Hongye Zhao, Mingjun Jiang, Yan Zhou, Dizhi Liu, Sihang Fang, Danni Gao, Zhaoping Wang, Ze Yang, Huiping Yuan

**Affiliations:** 1 Department of Clinical Laboratory, the Seventh Medical Center, Chinese PLA General Hospital, Beijing 100700, P.R. China.; 2 Department of Research & Development, Beijing IPE Center for Clinical Laboratory CO, Beijing 100176, P.R. China.; 3 Department of Biochemistry and Molecular Biology, The Key Laboratory of Neural and Vascular Biology, Ministry of Education of China, Hebei Medical University, Shijiazhuang, Hebei 050017, P.R. China.; 4 Respiratory Department, Beijing Children's Hospital, Capital Medical University, China National Clinical Research Center of Respiratory Diseases, National Center for Children's Health, Beijing 100045, P.R. China.; 5 General Practice Department, Beijing Hospital, Beijing 100730, P.R. China.; 6 The Key Laboratory of Geriatrics, Beijing Institute of Geriatrics, Institute of Geriatric Medicine, Chinese Academy of Medical Sciences, Beijing Hospital/National Center of Gerontology of National Health Commission, Beijing 100730, P.R. China.

**Keywords:** proteomics, aging, homeostasis, inflammation, metabolism, oxidative stress

## Abstract

As effectors of interactions between genes and the environment, plasma proteins can monitor homeostasis and reflect the aging state of an organism. However, biomarkers of aging that are associated with homeostasis are still unclear. This study investigates the phenotype-related plasma proteome profiles of healthy individuals and to identify proteins that are specifically related to aging and physiological indices and their expression patterns across the lifespan. From September 2020 to March 2021, 71 participants aged over 20 to 100 years were enrolled in this cross-sectional study. Data were analyzed from April 2021 to December 2023. The plasma proteome was analyzed to identify proteins that are specifically related to aging and their expression patterns across the lifespan. Then, hub proteins were screened through correlation of aging proteins with physiological and biochemical phenotypes. Based on levels of plasma proteins, physiological indices are associated with age. Additionally, these differences in protein expression correlate with age and physiological indices. Finally, we identified 20 hub proteins that correlate with both physiological indices and age, and these proteins are involved in oxidative stress, inflammation and metabolism. Bibliometric analysis confirmed that 8 hub proteins (CD44, CD14, IGF2, CFD, LBP, IGFBP3, EFEMP1, and AHSG) associated with age affect organ function by mediating homeostasis. Plasma proteins associated with both age and physiological indices are involved in oxidative stress, inflammation, and metabolism. This is the first investigation to link aging and homeostasis based on plasma proteins.

## Introduction

**Figure 1:**
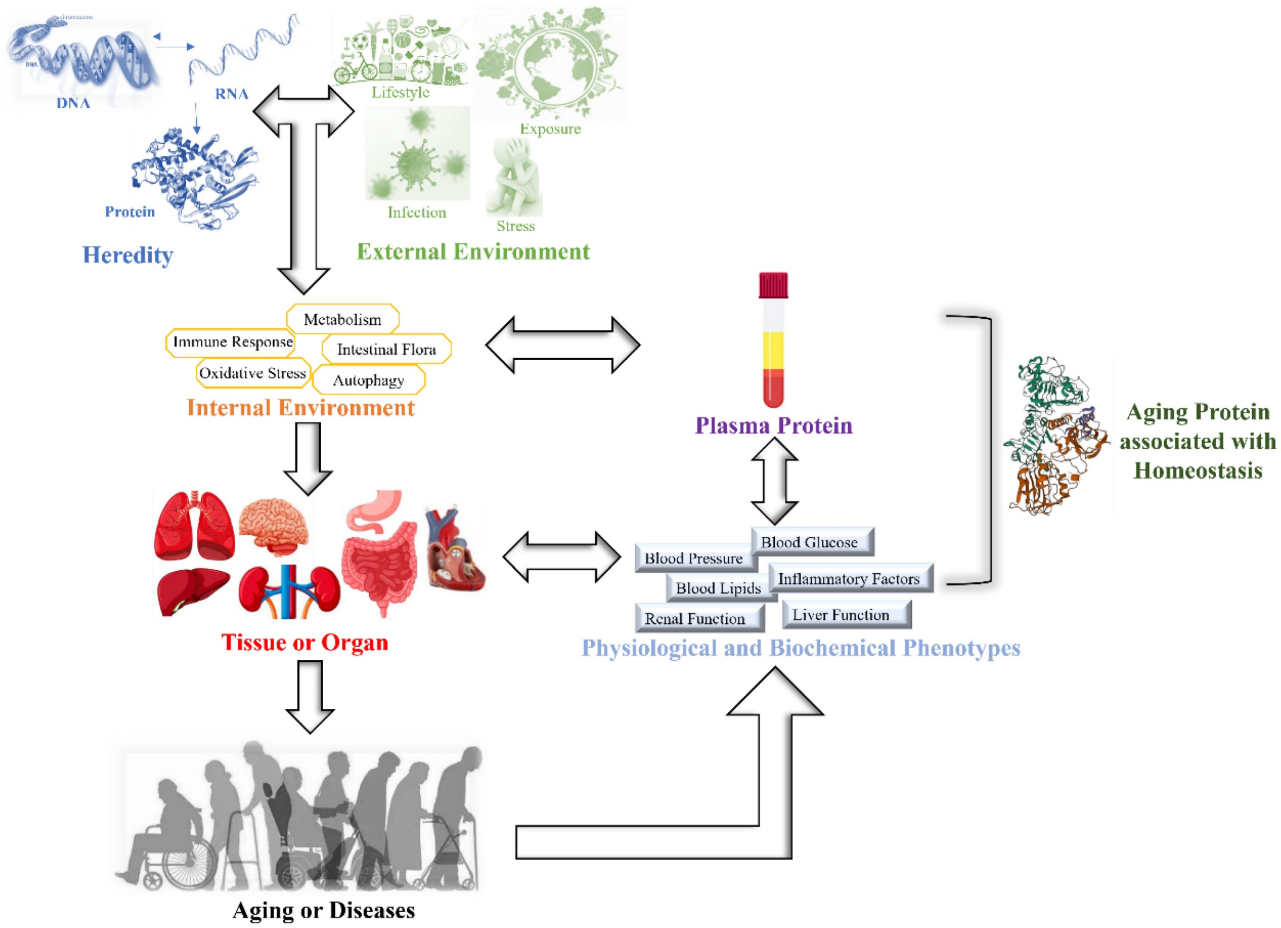
Schematic diagram of this study.

Aging is a time-dependent systematic degenerative physiological process that is influenced by interactions between genes and the environment 1, 2. The influence of interactions between genes and the environment on the body gradually increases with age, leading to an imbalance in the homeostasis of the internal environment. This induces a reduction or loss of tissue or organ function and results in a state of disease 3, 4. Aging and uncontrollable environmental factors are inevitable, but homeostasis can be effectively monitored by physiological indices. When the body is under external environmental pressure, a series of internal environmental regulatory molecules, such as inflammatory factors, metabolic factors and oxidative stress products, are activated to maintain internal environmental homeostasis 5 (Figure 1). Epidemiological studies have shown a marked increase in pro-inflammatory cytokines in aged individuals. Numerous studies have suggested that C-reactive protein (CRP) is connected with age-related conditions and diseases 6, 7. The immune system has evolved to initiate powerful and acute responses to effectively eliminate pathogens and protect tissue integrity 8. Therefore, CRP is an important factor reflecting immune inflammatory homeostasis and healthy aging.

Metabolism is a crucial and complex biochemical process involved in energy storage and the maintenance of normal biological functions 9, 10. Various interventions involving diet, drugs, genetics, and surgery that affect lipids can prolong the lifespan of model organisms. In humans, blood lipid levels, except high-density lipoprotein cholesterol (HDL-C), tend to increase with age. Blood lipids may be a rich source of biomarkers of aging in humans 11. Moreover, fasting blood glucose levels increase over the human lifespan. Higher glucose levels are associated with higher mortality, suggesting a link between blood glucose and aging 12.

Oxidative stress is generally considered to be the main mechanism that limits lifespan. Reactive oxygen species (ROS), which are the main byproducts of oxygen metabolism and adenosine triphosphate (ATP) production, can be successfully cleared by superoxide dismutase (SOD) and other radical scavengers. Therefore, SOD is an effective indicator that indirectly reflects ROS in the human body 13. When external stress causes ROS to exceed antioxidant capacity, oxidative stress is induced, which is directly related to the development of many diseases that limit healthy aging 14.

With the aging of the population, an increasing number of researchers have applied genomics, transcriptomics and proteomics to studies of aging. Proteins are direct effectors of cells, body fluids and tissues after gene modification and expression, reflecting changes in expression of genes affected by the environment. Aging leads to changes in protein composition, which helps to understand complex biological processes. Specifically, blood, which includes proteins from nearly all cells and tissues, has been studied to discover biomarkers and comprehend homeostasis 1, and plasma proteins can be separated from plasma through a feasible and convenient collection procedure 15. Therefore, plasma proteins can be used as convenient and accurate sources for identifying biomarkers of aging. However, biomarkers of aging that are associated with homeostasis are still unclear.

For this reason, our study aimed to determine proteins related to aging that have associations with physiological indices (blood pressure, blood glucose, blood lipids, CRP, SOD, liver function and renal function) in young individuals and centenarians to gain a preliminary understanding of homeostasis through these proteins. We employed a data-independent acquisition (DIA) liquid chromatography-tandem mass spectrometry (LC-MS/MS) technique to enhance protein coverage and minimize variation in sample preparation. By employing this technique alongside statistical analysis, we examined the plasma proteome to pinpoint proteins specifically associated with aging and their expression patterns throughout the lifespan. We subsequently correlated aging-related proteins with physiological and biochemical phenotypes to screen for hub proteins. Finally, we validated these hub proteins by integrating the findings of previous studies.

## Methods

### Study cohort

Seventy-one participants aged >20-100 years and without adverse outcomes 16 were recruited from September 2020 to March 2021. The patients were grouped according to 10-year intervals in an equal distribution of sample number and sex. The Ethics Committee of Beijing Hospital approved the study protocol (2019BJYYEC-118-02). The study was conducted in accordance with the Declaration of Helsinki and its amendments. All study participants (or their caregivers) provided written informed consent prior to enrollment.

### Sample preparation and protein extraction for DIA-seq

Using venipuncture, blood treated with EDTA was collected, and plasma was separated. The extraction of total proteins was performed with the cold acetone method. Samples were dissolved in 2 mL of lysis buffer containing 8 M urea, 2% sodium dodecyl sulfate, and a protease inhibitor cocktail from Roche Ltd., Switzerland. They underwent sonication on ice for 30 minutes and were then centrifuged at 13,000 rpm for 30 minutes at 4 °C. The supernatant was placed into a new tube, and proteins for each sample were precipitated with ice-cold acetone at -20 °C overnight. Following three acetone cleanings, the precipitates were redissolved in 8 M urea through sonication on ice. SDS‒PAGE (sodium dodecyl sulfate - polyacrylamide gel electrophoresis, SDS-PAGE) was employed to analyze the quality of the protein. Following the manufacturer's instructions, a BCA protein assay kit (Beyotime, China) was used to determine protein concentrations.

### High-pH reversed-phase fractionation

The protein sample was re-dissolved in buffer A, which consists of 20 mM ammonium formate in water at pH 10, adjusted with ammonium hydroxide, and then separated by high-pH fractionation using an Ultimate 3,000 system (Thermo Fisher Scientific, USA) linked to a reverse-phase column (XBridge C18 column, 4.6 mm × 250 mm, 5 μm; Waters Corporation, USA). A high-pH fractionation was conducted with a linear gradient ranging from 5% B to 45% B over 40 minutes, using 20 mM ammonium formate in 80% ACN at pH 10, adjusted with ammonium hydroxide. The column underwent re-equilibration to its original conditions for 15 minutes. The column's flow rate was consistently 1 mL/min, and the temperature was controlled at 30 °C. Ten fractions were obtained, and each was dried in a vacuum concentrator for further steps.

### Nanohigh-performance liquid chromatography‒mass tandem spectrometry (HPLC‒MS/MS) analysis

After being redissolved in 30 μL of solvent A (0.1% formic acid in water), the peptides were analyzed through on-line nanospray LC‒MS/MS with an Orbitrap Fusion Lumos linked to an EASY-nLC 1200 system (Thermo Fisher Scientific, USA). The analytical column (Acclaim PepMap C18, 75 μm × 25 cm) was loaded with a 3 μL peptide sample and separated over 120 minutes with a gradient from 5% to 35% B (0.1% formic acid in ACN). The column was maintained at a temperature of 40 °C with a flow rate of 200 μL/min. A 2 kV electrospray voltage was applied relative to the mass spectrometer's inlet. The mass spectrometer was set to data-independent acquisition mode, switching automatically between MS and MS/MS modes.

### Data analysis

Using default parameters, Spectronaut X (Biognosys AG, Switzerland) processed and analyzed the raw DIA data. For retention-time prediction, dynamic interactive response technology (iRT) was applied. Spectronaut X handled data extraction, utilizing extensive mass calibration. Spectronaut Pulsar X identified the optimal extraction window size based on iRT calibration and gradient stability, applying a 1% FDR cutoff at both precursor and protein levels. Decoy generation was configured to mutate, much like scrambling, but it was executed with a random number of amino acid (AA) position swaps (min = 2, max = length/2). Quantification was performed using all the selected precursors that passed through the filters. The major group quantities were calculated by averaging the top 3 filtered peptides that met the 1% Q-value threshold. A Student's t-test was conducted, and DEPs with a Q value < 0.05 and an absolute AVG log2 >0.58 were excluded. The extent of missing data was assessed using the 'mice' package in R version 4.0.3 (2020-10-10). For further analyses, we focused on proteins found in at least half of the samples. We used the R package gmodels (http://www.r-project.org/) to conduct principal component analysis (PCA). PCA is a statistical technique that changes a large number of correlated variables, like gene expression, into a set of linearly uncorrelated variables called principal components. It is mainly employed to identify relationships among samples.

### Age-related protein clustering

We conducted cluster analyses of age-related proteins to identify patterns linked to age. Age-related intervals were used to visualize trends.

1. Plots were divided into age groups of 0-40, 41-60, 61-80, and 81-100 years.

2. Plots for groups for an age span of 10 years each.

After calculating the mean protein intensity for each cluster, the average was taken for each age interval. The protein data were scaled for clustering, and Euclidean distance was used to calculate the distance between protein observations. The observations were clustered using complete linkage.

### Age-related proteins

The “limma” R package was used for differential expression analyses. To study the proteins with differential abundance, the following models were utilized: *Basic model* protein ~ Age.

### Waves of age-related proteins

Differential expression sliding window analysis (DEswan ^1^) was used to identify waves of aging plasma proteins. Using the DEswan function from the DEswan package, an age span of 20 to 100 years with 10-year intervals was chosen for sliding window analyses. In these DEswan analyses, sex was included as a covariate.

Considering a vector l of k unique ages, we iteratively used l_k_ as the age and compared the protein levels of individuals in parcels below and above l_k_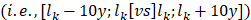. To test for differential expression, we used the following linear model:



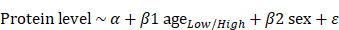



with age binarized according to the parcels. For each l_k_, q values were estimated using the Benjamini-Hochberg correction. Using the ANOVA function in the R package, the type II sum of squares was determined. A volcano plot was produced utilizing the R package 'EnhancedVolcano'.

### UpSet plot and gene interaction network construction

The UpSet technique provides a new way to visualize and quantitatively analyze interaction sets 17. We used these data to analyze the intersection between groups with an age span of 10 years each. The R package “ComplexHeatmap” was used to perform the analysis and visualization.

### Scatter plots and Circos plots showing correlations between proteins and phenotypes

The screening method used for physiological index-related proteins was the same as that used for age-related proteins. Figures were created in the R statistical software environment using two graphics packages, ggplot2 and circ.plot. The threshold for statistical significance was p < 0.05.

### Bibliometric analysis

We conducted a literature search of the Web of Science database in July 2023. The search terms used were [(“AHSG” OR “APOA2” OR “BCHE” OR “VCAM1” OR “CD14” OR “SERPINA3” OR “CD44” OR “ORM1” OR “CFD” OR “LRG1” OR “LBP” OR “CNDP1” OR “CST3” OR “EFEMP1” OR “ITIH3” OR “IGFBP3” OR “IGFALS” OR “FETUB” OR “GPLD1” OR “IGF2”) AND (“aging”)]. A total of 7,069 items that met the search criteria were found and analyzed further. Synonymous keywords and search terms, which affect the analysis results of research hotspots, were avoided. Generic words equivalent to research were deleted first, and then the keywords that appeared as search terms were deleted. The synonymous keywords were combined, and finally, the keywords with word frequencies greater than 15 were selected for mapping. Of the 24,791 keywords, 794 met the threshold. VOSviewer9 (version 1.6.18) was used to perform bibliometric analysis and visualization.

## Results

### Characteristics of the plasma proteome

**Figure 2:**
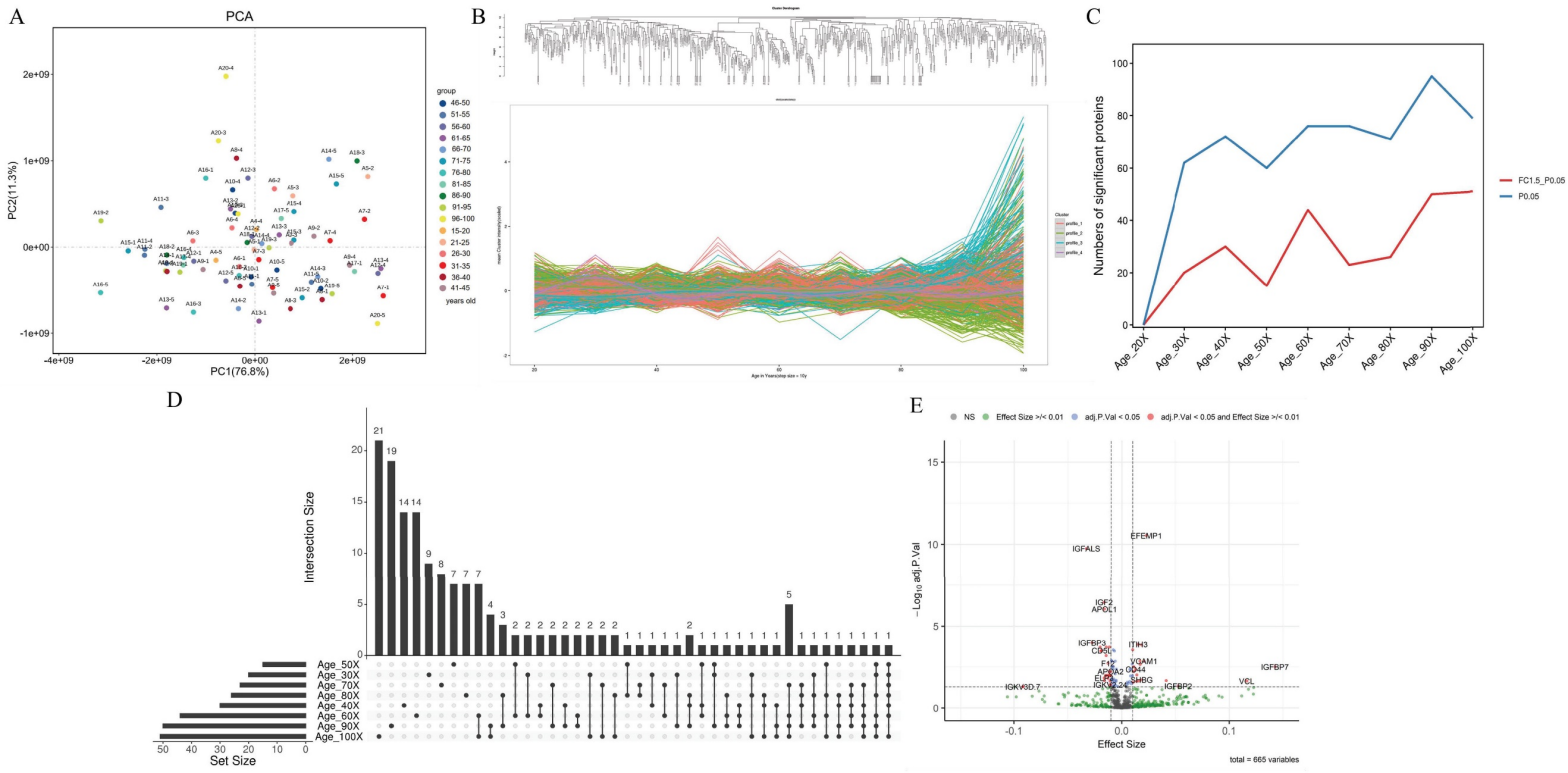
Plasma proteome profiling of healthy individuals with age. (A) Principal component analysis plot showing the distribution of each sample based on protein quantity. (B) Plasma protein clusters and their trajectories plotted against age in years (x-axis). (C) DEswan analysis of proteins at 10-year intervals. (D) UpSet plot depicting significant proteins at 10-year intervals (×30, ×40, ×50, ×60, ×70, ×80, ×90 and ×100). (E) Volcano plot depicting protein correlations with age. Red dots represent proteins that correlated significantly with age at an adjusted p <0.05 and an effect size cutoff >/<0.01. Blue dots represent significant proteins based only on an adjusted p value < 0.05. Green dots represent proteins for which the effect size cutoff was >/<0.01. Gray dots are proteins with expression that did not significantly change or with an effect size not significantly different.

A total of 71 plasma samples from healthy individuals aged >20 to 100 years were collected in our study. A total of 1,351 proteins were identified and quantified after analyzing the proteomic data through data-independent acquisition (DIA). After excluding proteins with more than 50% missing values across samples, the final list comprised 666 proteins (Table 1 in the Supplement). According to the protein expression data, the overall sample exhibited good clustering in the principal component analysis (PCA) plot (Figure 2A). The proportions of explained variance for PC1 and PC2 were 76.80% and 11.30%, respectively (88.10% in total).

### Clustering of plasma proteome trajectories

Genes were divided into four clusters according to the age interval 10 years (Figure 2B). Cluster 1 represented a group of conserved proteins (257 proteins) that did not change with age (Figure S1A in Supplement). Cluster 2 (238 proteins) showed a stepwise decreasing trajectory with age (Figure 1B in the Supplement), and Cluster 3 (152 proteins) showed a gradual increase with age (Figure S1C in the Supplement). Another 19 proteins in Cluster 4 exhibited irregular wave-like changes with age (Figure S1D in Supplement).

### Physiological indices associated with age based on protein expression

The characteristics of the physiological indices are shown in Figure S2 in the Supplement. According to the association rule algorithm and protein expression, age was the leading factor in the association rule. Physiological indices, including blood pressure, blood glucose, blood lipids, C-reactive protein (CRP), superoxide dismutase (SOD), liver function examination items and renal function examination items, were the consequents of the association rule. A total of 37 strong association rules were identified by the Apriori algorithm, with support and confidence thresholds of 72.73% and 100%, and a lift greater than 1. The results showed that FPG, DBIL, ALT, HDL, SOD, GOT, DBP, TBIL, and TC were strongly associated with the 30-year-old age group (Table 1). There were 7 indices (HDL, SOD, GOT, DBP, TC, Cr, and GGT) that could be used as predictors in the 40-year-old age group, with three indicators (DBP, TBIL, and SBP) as predictors of the 50-year age group and five indicators (HDL, SOD, GOT, TBIL, and SBP) as predictors of the 60-year-old age group.

### The correlation of plasma proteins with age

To better understand how the proteome changes with age, we employed the DEswan algorithm, which offers important insights into protein alterations at particular life stages. Overall, the number of significant proteins that changed increased with age. Three age groups with peaks at ages 40, 60 and 90 years were identified (Figure 2C). The UpSet plot also verified the number of significant proteins that were unique or shared between age groups (Figure 2D). There were 14 unique proteins related mainly to signal transduction at age 40 years (Table 2). There were also 14 and 19 unique proteins related mainly to metabolism at ages 60 and 90 years, respectively (Table 2). Two common proteins (integrin subunit alpha 2b, ITGA2B, and vinculin, VCL) were identified in the 40-, 80- and 90-year-old groups (Table 2). Five common proteins (carbonic anhydrase 1, CA1; hemoglobin subunit alpha 2, HBA2; hemoglobin subunit beta, HBB; hemoglobin subunit delta, HBD; and von Willebrand factor, VWF) were identified in the 60-, 70-, 90- and 100-year-old groups (Table 2). In addition to carbonic anhydrase 1 (CA1), which is associated with metabolism, common proteins are associated with the immune response.

To further discriminate between up- and downregulated proteins, the effect size was assessed using log2-transformed protein intensity. For every year of age, there was a 0.01 rise in the log-transformed protein intensity at this threshold. Twenty-six age-associated proteins were downregulated and 19 upregulated, as shown in a volcano plot (Figure 2E). Among these upregulated proteins, complement factor D (CFD), fibrinogen alpha chain (FGA), vascular cell adhesion molecule 1 (VCAM1), CD14 (CD14), CD44 (CD44), complement factor H related 2 (CFHR2), vinculin (VCL), and lipopolysaccharide-binding protein (LBP) are related to the immune response (Table 3). The downregulated proteins, except for several immunoglobulins and alpha 2-HS glycoprotein (AHSG) associated with the immune response, are related to metabolism and included insulin-like growth Factor 2 (IGF2), insulin-like growth factor binding protein acid labile subunit (IGFALS), insulin-like growth factor binding protein 3 (IGFBP3), apolipoprotein L1 (APOL1), butyrylcholinesterase (BCHE), carnosine dipeptidase 1 (CNDP1), apolipoprotein A2 (APOA2), glycosylphosphatidylinositol specific phospholipase D1 (GPLD1) and lecithin-cholesterol acyltransferase (LCAT) (Table 3).

### Correlations of proteins with both physiological indices and age

**Figure 3:**
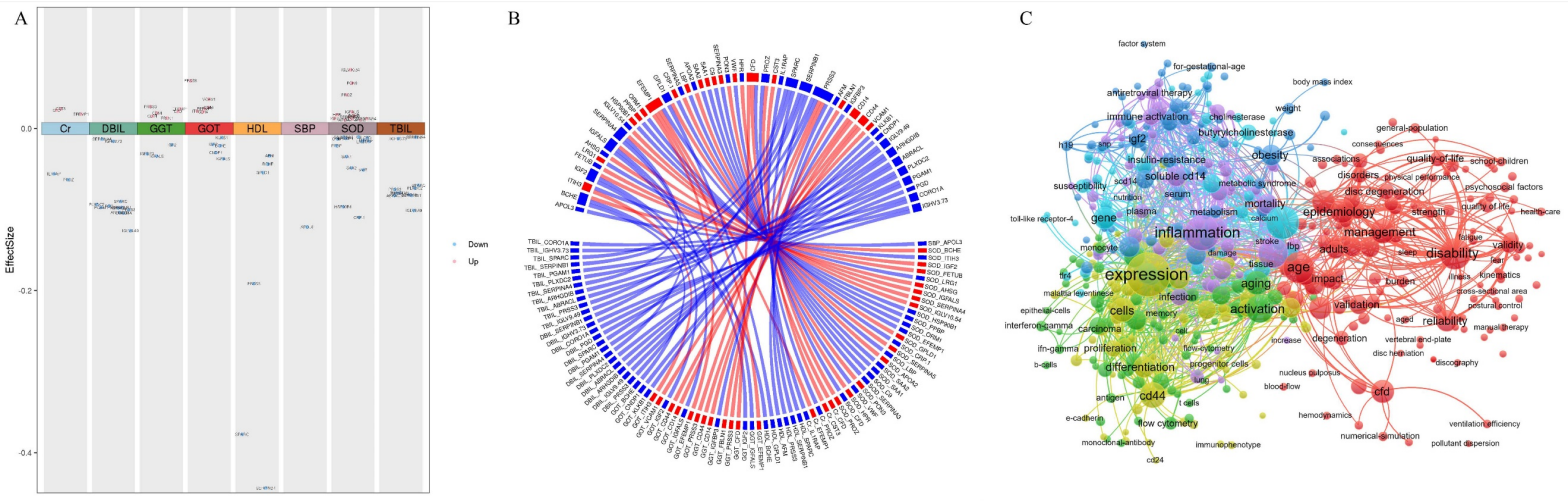
Phenotype-related plasma proteome profiling (A) Plasma proteins significantly associated with physiological indices. Red represents a positive correlation, and blue represents a negative correlation. (B) Circos plot showing correlations between plasma proteins and age and physiological indices and the association between the two variables. (C) The cluster map of keywords in proteomics and aging research. The color of the nodes denotes the cluster.

In the present dataset, only one protein, APOL3, which is a member of the apolipoprotein L gene family, correlated negatively with SBP but not with age (Figure 3A, 3B and Table S2 in the Supplement). Twenty-seven proteins correlated significantly with SOD activity, including 14 proteins that correlated negatively and 13 proteins that correlated positively with SOD activity (Figure 3A and Table S2 in the Supplement). Among these proteins, 14 proteins (BCHE, ITIH3, IGF2, FETUB, LRG1, AHSG, IGFALS, ORM1, EFEMP1, GPLD1, LBP, APOA2, SERPINA3, and CFD) correlated significantly with age but showed the opposite correlation with SOD (Figure 3B). Four proteins, but not PROZ, correlated positively with Cr (Figure 3A and eTable 2 in the Supplement). Among these 4 proteins, CFD, CST3 and EFEMP1 correlated significantly with age, and the correlation was consistent with that of Cr (Figure 3B). All 6 proteins correlated negatively with HDL cholesterol (Figure 3A and Table S2 in the Supplement). Among these 6 proteins, both GPLD1 and BCHE also correlated significantly negatively with age. Nine proteins correlated significantly with GGT levels, including 3 proteins that correlated negatively and 6 proteins that correlated positively with GGT levels (Figure 3A and Table S2 in the Supplement). PRSS3 and FBLN1 correlated positively with GGT. However, the other six proteins (EFEMP1, IGFALS, IGF2, CFD, IGFBP3, CD14, and CD44) correlated significantly with age, and these correlations were consistent with those for GGT (Figure 3B). Eleven proteins correlated significantly with GOT, including 5 proteins that correlated negatively and 6 proteins that correlated positively with GOT (Figure 3A and Table S2 in the Supplement). Nine proteins (EFEMP1, IGFALS, CD14, CD44, IGF2, VCAM1, ITIH3, CNDP1, and BCHE), but not PRSS3 or KLKB1, correlated significantly with age, and the correlation was consistent with that of GOT (Figure 3B). All 12 proteins and 11 proteins correlated negatively with DBIL and TBIL, respectively, but did not correlate with age (Figure 3A, 3B and Table S2 in the Supplement).

### Hub proteins involved in homeostasis

Based on the correlation analysis, 20 proteins were identified as hub proteins (BCHE, ITIH3, IGF2, FETUB, LRG1, AHSG, IGFALS, ORM1, EFEMP1, GPLD1, LBP, APOA2, SERPINA3, CFD, CST3, IGFBP3, CD14, CD44, VCAM1, and CNDP1) that correlate with both physiological indices and age. As of July 2023, there were 7,069 articles related to the hub proteins associated with aging, including 198 reviews and 6,871 articles. Keywords denote the main theme of a paper, and analyzing their co-occurrence can quickly highlight trending research topics in a certain sector. The high frequency of occurrence of hub proteins associated with aging (CD44, CD14, IGF2, CFD, LBP, IGFBP3, EFEMP1, and AHSG) and the top 20 high-frequency keywords are shown in Table S3 in the Supplement. Among them, inflammation occurred 490 times; thus, inflammation is a hot topic for identifying the hub proteins involved in aging and homeostasis. Disease, health, age and aging were keywords that closely followed inflammation. The sixth most common keyword was obesity, which also involves homeostasis. Although oxidative stress was low on the list, it also had a place on the top list. Using VOSviewer, the keywords were analyzed and visualized (Figure 3C), with a minimum threshold of 15 occurrences.

The identification of protein clusters has significant implications for understanding specific physiological processes. Protein clustering is a fundamental aspect of cellular organization, providing an intermediate level of structure between individual molecules and the larger cellular architecture. These clusters can influence a variety of cellular functions, including signal transduction, cellular communication, and metabolic regulation. Moreover, protein clusters are integral to the regulation of gene expression and cellular dynamics. These clusters have the potential to affect processes such as RNA metabolism and splicing, which are essential for preserving cellular homeostasis and functionality. In our study, a total of 295 keywords were identified, and they were divided into 6 clusters of different colors representing different research directions. The keywords in the red cluster are related mainly to age and health, including age, adulthood, disability, and disorders. The keywords in the green cluster are related mainly to immunity, including antigen, antibody, activation, and monocyte. The keywords in the blue cluster are related mainly to metabolism, including obesity, metabolic syndrome, and insulin resistance. The keywords in the yellow cluster are related mainly to cell differentiation and expression, including expression, differentiation and proliferation. The keywords in the purple cluster are related mainly to diseases, including inflammation, infection, stroke, and cholinesterase. The keywords in the cyan cluster are related mainly to metabolic proteases, including AHSG, butyrylcholinesterase, apolipoprotein-e, and fetuin-a.

## Discussion

The interaction between genetic and environmental factors alters the homeostasis of cells or tissues, which, with cumulative effects over time and dose, leads to adaptive changes in the structure and function of the body's organs and systems, as reflected in alterations at the physiological and biochemical levels and ultimately leading to an aging phenotype^18^. Because of the multifactorial process of aging, devising effective explanations of senescence as a whole is a challenge^19^.

Studies have shown that homeostasis can be monitored by plasma proteins, and due to the cumulative effect on physiological and biochemical indicators, these proteins can ultimately reflect the aging phenotype 20-23 (Figure 1). However, it is unclear which plasma proteins can be used as biomarkers of homeostasis and influence aging.

Recent studies have shown that plasma proteins are differentially expressed across the lifespan 1. Four protein clusters were found in our study, and these clusters exhibited different expression trends with age (Figure 2B). Furthermore, we identified 45 age-related proteins. The proteins that were upregulated with age are related mainly to the immune response, whereas the proteins that were downregulated with age are related mainly to metabolism (Table 3). As demonstrated in previous studies, the increase in the immune response with age is reflected in the increased susceptibility to infectious diseases and increased prevalence of chronic diseases characterized by a pro-inflammatory state 24, and basal energy metabolism is thought to decline linearly with age 25. Three age groups with peaks at 40, 60 and 90 years were identified (Figure 2C). The unique proteins at ages 60 and 90 years are mainly related to metabolism. Common proteins of the 60-, 70-, 90- and 100-year-old groups are mainly associated with the immune response and metabolism (Table 2). A large number of studies have also suggested that metabolic balance is a crucial requirement for cellular homeostasis. Changes associated with aging stimulate the innate immune system, leading to low-level inflammation and metabolic disorders 26. Our results confirm the findings of previous studies.

Several biochemical indices have a strong connection with physical function, morbidity and mortality, indicating that the changes in these indices are related to health and indirectly reflected the aging process 27, 28. Our protein expression data showed that different biochemical indices are reflected in different age intervals (Table 1). By further identifying and validating proteins that correlate with physiological indices and age, we found that aging-related studies have linked mainly CD44, CD14, IGF2, CFD, LBP, IGFBP3, EFEMP1, and AHSG to inflammation and metabolism. Among them, CD44, CD14, IGF2, CFD, and EFEMP1 are mainly associated with liver function; CFD and EFEMP1 are mainly associated with renal function; and IGF2, CFD, LBP, EFEMP1, and AHSG are mainly associated with oxidative stress.

### Hub proteins associated with liver function influence healthy aging through homeostasis

CD44 is a cell membrane-bound surface receptor that mediates inter-cellular and extracellular matrix (ECM) communication 29. It is considered a potential biomarker of aging in healthy brains through immune inflammatory responses 30, 31. In addition, CD44 is involved in the relationship between hepatocyte lipotoxicity and inflammatory cell infiltration 32, which suggests a potential link between CD44 and lipid metabolism. CD14 is a membrane glycoprotein anchored by glycosylphosphotidylinositol, found on neutrophils and mononuclear cells/macrophages, and also exists in a soluble form known as sCD14 33, 34. CD14 plays a key role in the chronic inflammatory response in elderly people and chronic kidney disease (CKD) patients and has a direct relationship with the development of CVD 35. High levels of circulating soluble CD14 (sCD14) mediate the onset and development of atherosclerosis by stimulating macrophages to produce pro-inflammatory molecules 36. As for other acute-phase inflammatory biomarkers, sCD14 predicts the onset of CVD and independently predicts all-cause mortality in older adults 37. According to extensive literature, CD14 and CD44 play important roles in adipose tissue inflammation, which in turn enhances insulin resistance and hepatic function damage and even affects aging 38, 39. CD44 interacts with the PI3K-AKT-mTOR pathway, which is crucial for cell survival, growth, and metabolism, thereby influencing cellular aging and homeostasis 40. CD14's regulation of inflammation is vital for homeostasis and preventing aging-related chronic inflammation 41. Our results also confirmed the associations between CD14/CD44 and GGT/GOT, which are indicators of liver function, and age (Figure 3B).

IGF2, a member of the insulin family of polypeptide growth factors, is involved in glucose metabolism in adipose tissue, the liver, and aging 42-44. Research has demonstrated that higher IGF2 levels enhance memory in healthy animals and mitigate numerous symptoms in laboratory aging models 45. It influences cell growth and specialization, with its imbalance linked to diseases like cancer and metabolic disorders. IGF2 engages with the IGF pathway, affecting aging and lifespan by regulating cell growth and metabolism 46. This was confirmed by the correlation between IGF2 and GOT/GGT, which are representative indicators of liver function or age (Figure 3B), in our study.

EFEMP1 is a member of the fibulin family of extracellular matrix glycoproteins. Studies have shown that elevated levels of circulating EFEMP1 are associated with an increased risk of all-cause and AD dementia 47. It may influence cognitive decline through its effects on brain structure and function, highlighting its role in aging and homeostasis 48. Efemp1 knockout mice were reported to age faster and die earlier than wild-type mice did, and they displayed early aging phenotypes 49. EFEMP1 is also a crucial gene involved in the development of nonalcoholic steatohepatitis (NASH) via extracellular matrix (ECM)-related pathways or immunity-related pathways 50. It has been suggested that EFEMP1 correlates with liver function, which is consistent with our results (Figure 3B).

Complement factor D (CFD), a type of serine protease, facilitates the cleavage of complement factor B, which is the rate-limiting step in the alternative complement activation pathway 51. CFD activates the alternative complement pathway in the immune system, influencing inflammation and immune surveillance. Its dysregulation can contribute to age-related diseases and disrupt tissue homeostasis. This protein also functions as an adipokine. Adipocyte differentiation and lipid accumulation are influenced by CFD via C3a signaling 52. Our results revealed an association between CFD and an indicator of liver function (GGT) (Figure 3B). However, the specific molecular mechanisms of CFD in organ damage and aging need to be further explored.

Insulin-like Growth Factor Binding Protein 3 (IGFBP-3) is a member of the IGFBP family. IGFBP3 modulates the activity of IGF1 and IGF2 by binding to them and regulating their interaction with IGF receptors. It plays a role in cell growth, survival, and apoptosis, and its expression is associated with aging and age-related diseases. IGFBP3 can influence the IGF signaling pathway, which is critical for regulating cellular aging and homeostasis 53. A study on the relationship between the insulin-like growth factor axis and plasma lipid levels in elderly individuals revealed that age was inversely associated with IGFBP-3 levels, body mass index, and lipid levels. IGFBP-3 correlates significantly positively with HDL-C and ApoA1 54. In our study, the plasma level of IGFBP-3 correlated significantly with the level of GGT, which is associated with lipid metabolism (Figure 3B). Animal experiments have shown that overexpression of human IGFBP-3 or its mutant devoid of IGF-binding ability leads to glucose intolerance with different effects on insulin secretion, insulin sensitivity, and lipid homeostasis in aging mice 55. Therefore, IGFBP-3 is closely related to liver function and lipid homeostasis.

### Hub proteins associated with renal function influence healthy aging through homeostasis

According to previous studies, EFEMP1 is an extracellular matrix protein involved in both cellular structure and signaling. Additionally, it was associated with worse eGFR cross-sectionally, with a longitudinal ΔeGFR, with prevalent chronic kidney disease (CKD), and with a rapid decrease in eGFR. A study also confirmed that there is a putative causal relationship between the EFEMP1 concentration and the estimated glomerular filtration rate (eGFR), suggesting a relationship between EFEMP1 and renal function 56. In addition, it has been shown that higher levels of circulating CFD are associated with a lower risk of developing diabetes in middle-aged adults 57, and deficiency in complement factor D is closely related to inflammation 58. Patients with chronic kidney disease (CKD) have been found to have high levels of CFD in plasma microparticles 59. Inhibiting CFD may help to prevent amplification of the alternative pathway and thereby limit systemic inflammation, organ damage, and disease progression 51. Therefore, in addition to liver function, kidney function is related to both EFEMP1 and CFD.

### Hub proteins associated with SOD influence healthy aging through homeostasis

A recent report on adult neuronal culture-derived cell lines demonstrated that IGF2 increases mitochondrial functional activity by reducing oxidative stress, which affects aging 60. This was confirmed by the correlation between IGF2 and SOD, which are representative indicators of oxidative stress (Figure 3B), in our study. These results reveal the key role of IGF2 in homeostasis and aging; thus, IGF2 may serve as a promising biomarker for predicting physical health and aging.

In addition to its effect on liver and kidney function, EFEMP1 (fibulin-3) is able to alleviate the changes in ROS levels in the low-dose fibulin 3 groups. Studies have also indicated that fibulin-3 may reduce the level of oxidative stress during hypertensive vascular remodeling 61, 62. Moreover, fibulin-3 may be related to oxidative stress, as indicated by our results (Figure 3B).

Furthermore, CFD has the potential to prevent oxidative stress-induced cell death without evident toxicity 63. Our results revealed an association between CFD and an indicator of oxidative stress (SOD) (Figure 3B). However, the specific molecular mechanisms of CFD in organ damage and aging need to be further explored.

Lipopolysaccharide-binding protein-1 (LBP) is the main component of the outer membrane of gram-negative bacteria. It can increase production of various inflammatory cytokines and chemokines and subsequently induce innate immunity in the liver 64. LBP aids the immune response to bacterial infections by binding LPS, enabling its detection by CD14 and Toll-like receptors, which initiate inflammation. While crucial for pathogen removal, this can lead to chronic inflammation and aging if unchecked 41. A relationship between and physical function has been reported in healthy older adults. Epidemiological analysis revealed that LBP-1 was positively associated with inflammatory factors and that LBP-1 was negatively associated with physical function 65. Furthermore, LBP mediates lysosomal signaling, which acts in parallel to regulating longevity 66. Current findings suggest that a significant decrease in liver LBP levels promotes liver oxidative stress and inflammation, aggravating nonalcoholic steatohepatitis (NASH) progression under physiological and pathological nonobesogenic conditions 67. Therefore, LBP is involved not only in inflammation and metabolism but also in oxidative stress and affects lifespan.

AHSG (alpha-2-HS-glycoprotein, fetuin-A), a liver-derived plasma protein, modulates inflammation, reduces insulin sensitivity, and promotes weight gain following a high-fat diet or aging 68. Clinical studies have shown that the serum fetuin A concentration correlates with age and increases with age 69. Moreover, low levels of fetuin-A may promote crystal deposition and subsequently induce cell injury and oxidative stress 70. Nevertheless, further investigations in elderly individuals are needed to validate the correlations between LBP and fetuin-A levels and oxidative stress and age found in our study, and subsequent functional studies may help to clarify the role of LBP and fetuin-A. AHSG's interaction with FGF23 suggests a role in modulating bone and vascular pathology, which are important aspects of aging and homeostasis.

## Conclusions

To conclude, in the present global plasma proteomic study of a cohort of 71 healthy individuals (aged>20-100), we identified and quantified 666 proteins. Different bioinformatics approaches were used to investigate proteins that correlated significantly with age and physiological indices. Furthermore, the identified hub proteins associated with both age and physiological indices are involved in oxidative stress, inflammation, and metabolism. To our knowledge, this is the first investigation to link aging and homeostasis based on plasma proteins. However, the specific molecular mechanisms underlying the role of these proteins in homeostasis and aging need to be further explored and validated in future studies.

## Supplementary Material

Supplementary figures and tables.

## Figures and Tables

**Table 1:** Physiological indexes associated with age.

Antecedents	Consequents	Antecedent support	Consequent support	Support	Confidence	Lift
age30	FPG	0.149	0.797	0.135	0.909	1.140
	DBIL	0.149	0.851	0.135	0.909	1.068
	ALT	0.149	0.851	0.135	0.909	1.068
	HDL	0.149	0.622	0.122	0.818	1.316
	SOD	0.149	0.649	0.122	0.818	1.261
	GOT	0.149	0.689	0.122	0.818	1.187
	DBP	0.149	0.757	0.122	0.818	1.081
	TBIL	0.149	0.811	0.122	0.818	1.009
	TC	0.149	0.608	0.108	0.727	1.196
age40	TC	0.135	0.608	0.122	0.900	1.480
	SOD	0.135	0.649	0.122	0.900	1.388
	DBP	0.135	0.757	0.122	0.900	1.189
	Cr	0.135	0.892	0.122	0.900	1.009
	GGT	0.135	0.595	0.108	0.800	1.345
	HDL	0.135	0.622	0.108	0.800	1.287
	GOT	0.135	0.689	0.108	0.800	1.161
age50	TBIL	0.135	0.811	0.122	0.900	1.110
	SBP	0.135	0.865	0.122	0.900	1.041
	DBP	0.135	0.757	0.108	0.800	1.057
age60	TBIL	0.135	0.811	0.122	0.900	1.110
	SBP	0.135	0.865	0.122	0.900	1.041
	HDL	0.135	0.622	0.108	0.800	1.287
	SOD	0.135	0.649	0.108	0.800	1.233
	GOT	0.135	0.689	0.108	0.800	1.161

**Table 2:** Protein changes at specific stages of life.

Age group	Protein	Description	KEGG_A_class	KEGG_B_class
Age_40×	ABI3BP	ABI family member 3 binding protein	-	-
	ACTG1	actin gamma 1	Human Diseases; Cellular Processes; Organismal Systems; Environmental Information Processing	Transport and catabolism; Infectious diseases; Cardiovascular diseases; Cellular community - eukaryotes; Cell motility; Cancers; Cell growth and death; Immune system; Signal transduction; Infectious disease: bacterial; Endocrine system; Environmental adaptation; Digestive system
	CAP1	cyclase associated actin cytoskeleton regulatory protein 1	-	-
	FLNA	filamin A	Cellular Processes; Human Diseases; Environmental Information Processing	Cellular community - eukaryotes; Cancers; Infectious diseases; Signal transduction
	HSPA8	heat shock protein family A (Hsp70) member 8	Cellular Processes; Organismal Systems; Environmental Information Processing; Genetic Information Processing	Transport and catabolism; Immune system; Signal transduction; Folding, sorting and degradation; Infectious diseases; Endocrine system; Transcription; Aging
	ICAM2	intercellular adhesion molecule 2	Environmental Information Processing; Organismal Systems	Signaling molecules and interaction; Immune system
	IGLV8-61	-	-	-
	KRT10	keratin 10	Human Diseases; Organismal Systems	Infectious diseases; Endocrine system
	LDHB	lactate dehydrogenase B	Metabolism; Environmental Information Processing; Human Diseases; Organismal Systems	Global and overview maps; Carbohydrate metabolism; Signal transduction; Cancers; Endocrine system; Amino acid metabolism
	LTBP1	latent transforming growth factor beta binding protein 1	Environmental Information Processing	Signal transduction
	RAP1B	RAP1B, member of RAS oncogene family	Cellular Processes; Organismal Systems; Environmental Information Processing; Human Diseases	Cellular community - eukaryotes; Immune system; Signal transduction; Digestive system; Nervous system; Endocrine and metabolic diseases; Cancers
	TF	transferrin	Environmental Information Processing; Cellular Processes; Organismal Systems	Signal transduction; Cell growth and death; Digestive system
	TNXB	tenascin XB	Environmental Information Processing; Cellular Processes; Human Diseases	Signal transduction; Cellular community - eukaryotes; Signaling molecules and interaction; Infectious diseases; Cancers
	YWHAZ	tyrosine 3-monooxygenase/tryptophan 5-monooxygenase activation protein zeta	Environmental Information Processing; Human Diseases; Cellular Processes	Signal transduction; Cancers; Cell growth and death; Infectious diseases
Age_60×	APMAP	adipocyte plasma membrane associated protein	-	-
	CLC	Charcot-Leyden crystal galectin	-	-
	ENO1	enolase 1	Metabolism; Environmental Information Processing; Genetic Information Processing	Global and overview maps; Carbohydrate metabolism; Signal transduction; Folding, sorting and degradation
	ESD	esterase D	-	-
	GAPDH	glyceraldehyde-3-phosphate dehydrogenase	Metabolism; Human Diseases; Environmental Information Processing	Global and overview maps; Infectious diseases; Neurodegenerative diseases; Carbohydrate metabolism; Signal transduction
	IGHV3-16	-	-	-
	IGKV2-30	-	-	-
	MIF	macrophage migration inhibitory factor	Metabolism	Global and overview maps; Amino acid metabolism
	MSN	moesin	Cellular Processes; Human Diseases; Organismal Systems	Cell motility; Cancers; Immune system; Cellular community - eukaryotes; Infectious diseases
	PFN1	profilin 1	Human Diseases; Cellular Processes; Environmental Information Processing	Infectious diseases; Cell motility; Signal transduction
	PNP	purine nucleoside phosphorylase	Metabolism	Global and overview maps; Nucleotide metabolism; Metabolism of cofactors and vitamins
	SH3BGRL3	SH3 domain binding glutamate rich protein like 3	-	-
	TXN	thioredoxin	Human Diseases; Organismal Systems	Infectious diseases; Immune system; Cardiovascular diseases
	YWHAB	tyrosine 3-monooxygenase/tryptophan 5-monooxygenase activation protein beta	Environmental Information Processing; Human Diseases; Cellular Processes	Signal transduction; Cancers; Cell growth and death; Infectious diseases
Age_90×	APOE	apolipoprotein E	Human Diseases; Organismal Systems	Neurodegenerative diseases; Digestive system
	B2M	beta-2-microglobulin	Human Diseases; Organismal Systems	Infectious diseases; Immune system
	CALR	calreticulin	Cellular Processes; Human Diseases; Organismal Systems; Genetic Information Processing	Transport and catabolism; Infectious diseases; Immune system; Folding, sorting and degradation
	CNDP1	carnosine dipeptidase 1	Metabolism	Global and overview maps; Amino acid metabolism; Metabolism of other amino acids
	FAH	fumarylacetoacetate hydrolase	Metabolism	Global and overview maps; Amino acid metabolism
	FCGR3A	Fc fragment of IgG receptor IIIa	Human Diseases; Cellular Processes; Organismal Systems	Immune diseases; Transport and catabolism; Infectious diseases; Immune system; Development
	G6PD	glucose-6-phosphate dehydrogenase	Metabolism; Human Diseases	Global and overview maps; Carbohydrate metabolism; Metabolism of other amino acids; Cancers
	GPLD1	glycosylphosphatidylinositol specific phospholipase D1	Metabolism	Global and overview maps; Glycan biosynthesis and metabolism
	IGF2	insulin like growth factor 2	Environmental Information Processing; Human Diseases	Signal transduction; Cancers
	IGFALS	insulin like growth factor binding protein acid labile subunit	Organismal Systems	Endocrine system
	IGHA2	immunoglobulin heavy constant alpha 2 (A2m marker)	Human Diseases; Environmental Information Processing; Cellular Processes; Organismal Systems	Immune diseases; Signal transduction; Transport and catabolism; Infectious diseases; Cardiovascular diseases; Immune system; Cancers
	LYVE1	lymphatic vessel endothelial hyaluronan receptor 1	-	-
	PCOLCE	procollagen C-endopeptidase enhancer	-	-
	SEMA4B	semaphorin 4B	Organismal Systems	Development
	TAGLN2	transgelin 2	-	-
	TGFBI	transforming growth factor beta induced	-	-
	VCAM1	vascular cell adhesion molecule 1	Environmental Information Processing; Human Diseases; Organismal Systems	Signaling molecules and interaction; Infectious diseases; Immune system; Signal transduction; Cardiovascular diseases; Endocrine and metabolic diseases
	WDR1	WD repeat domain 1	-	-
	ZYX	zyxin	Cellular Processes	Cellular community - eukaryotes
Age_40×_80×_90×	ITGA2B	integrin subunit alpha 2b	Environmental Information Processing; Human Diseases; Cellular Processes; Organismal Systems	Signal transduction; Cardiovascular diseases; Cellular community - eukaryotes; Cancers; Immune system; Cell motility; Signaling molecules and interaction; Infectious diseases
	VCL	vinculin	Human Diseases; Cellular Processes; Organismal Systems	Infectious diseases; Cellular community - eukaryotes; Cell motility; Immune system
Age_60×_70×_90×_100×	CA1	carbonic anhydrase 1	Metabolism	Global and overview maps; Energy metabolism
	HBA2	hemoglobin subunit alpha 2	Human Diseases	Infectious diseases
	HBB	hemoglobin subunit beta	Human Diseases	Infectious diseases
	HBD	hemoglobin subunit delta	-	-
	VWF	von Willebrand factor	Environmental Information Processing; Cellular Processes; Organismal Systems; Human Diseases	Signal transduction; Cellular community - eukaryotes; Immune system; Signaling molecules and interaction; Infectious diseases

**Table 3:** Proteins associated with age.

Protein	EffectSize	AveExpr	t	P.Value	adj.P.Val	B	Sig
EFEMP1	0.023	19.318	9.336	4.389E-14	2.918E-11	21.366	Up
ITIH3	0.015	21.520	5.299	1.185E-06	1.314E-04	4.413	Up
CFD	0.019	19.049	5.240	1.498E-06	1.423E-04	4.184	Up
FGA	0.010	27.256	4.951	4.612E-06	2.788E-04	3.087	Up
VCAM1	0.021	16.362	4.381	3.880E-05	0.001	1.021	Up
LRG1	0.017	22.611	4.364	4.135E-05	0.001	0.959	Up
CST3	0.017	19.691	4.187	7.797E-05	0.002	0.348	Up
IGFBP7	0.143	6.961	4.092	1.170E-04	0.003	0.015	Up
CD14	0.011	19.467	4.045	1.285E-04	0.003	-0.132	Up
CD44	0.013	18.668	3.910	2.050E-04	0.004	-0.579	Up
SERPINA3	0.011	25.506	3.791	3.067E-04	0.006	-0.963	Up
CFHR2	0.014	19.939	3.626	5.291E-04	0.009	-1.482	Up
SHBG	0.019	19.612	3.315	0.001	0.019	-2.418	Up
PCOLCE	0.041	16.489	3.256	0.002	0.021	-2.588	Up
SERPINA3_1	0.016	18.083	3.237	0.002	0.022	-2.643	Up
VCL	0.116	11.115	3.223	0.002	0.022	-2.680	Up
LBP	0.014	21.549	3.184	0.002	0.024	-2.795	Up
ORM1	0.010	27.227	3.118	0.003	0.027	-2.980	Up
IGFBP2	0.053	15.528	2.903	0.005	0.045	-3.562	Up
IGFALS	-0.033	20.389	-8.756	5.354E-13	1.780E-10	18.872	Down
IGF2	-0.016	20.236	-6.904	1.570E-09	3.481E-07	10.940	Down
APOL1	-0.016	20.987	-6.607	5.527E-09	9.189E-07	9.695	Down
IGFBP3	-0.028	20.337	-5.405	7.804E-07	1.038E-04	4.822	Down
BCHE	-0.014	20.101	-5.112	2.475E-06	1.928E-04	3.694	Down
AHSG	-0.012	26.196	-5.092	2.669E-06	1.928E-04	3.620	Down
IGHM	-0.020	26.980	-5.071	2.900E-06	1.928E-04	3.539	Down
CD5L	-0.019	23.299	-4.878	6.097E-06	3.119E-04	2.815	Down
CNDP1	-0.019	19.758	-4.825	7.454E-06	3.305E-04	2.619	Down
FETUB	-0.015	20.286	-4.636	1.518E-05	6.308E-04	1.929	Down
F12	-0.013	21.280	-4.251	6.214E-05	0.002	0.567	Down
APOA2	-0.010	27.621	-3.792	3.052E-04	0.006	-0.958	Down
IGKV1.17	-0.012	21.374	-3.764	3.355E-04	0.006	-1.049	Down
IGLV2.8	-0.012	21.234	-3.603	5.704E-04	0.010	-1.553	Down
IGKV2D.28	-0.010	23.622	-3.574	6.278E-04	0.010	-1.643	Down
IGLV2.18	-0.014	20.745	-3.572	6.319E-04	0.010	-1.650	Down
IGKV1.16	-0.015	20.623	-3.556	6.663E-04	0.010	-1.700	Down
TTR	-0.012	22.022	-3.547	6.860E-04	0.010	-1.727	Down
ELP3	-0.016	20.868	-3.429	9.984E-04	0.014	-2.082	Down
GPLD1	-0.013	20.140	-3.407	0.001	0.015	-2.149	Down
LCAT	-0.016	20.336	-3.259	0.002	0.021	-2.580	Down
IGKV1.5	-0.011	22.617	-3.228	0.002	0.022	-2.669	Down
IGKV2.24	-0.011	21.839	-2.989	0.004	0.037	-3.334	Down
IGKV2D.24	-0.011	21.839	-2.989	0.004	0.037	-3.334	Down
IGKV3D.7	-0.092	13.362	-2.850	0.006	0.049	-3.701	Down
IGKV3OR2.268	-0.092	13.362	-2.850	0.006	0.049	-3.701	Down

## Abbreviations

CRP: C-reactive protein
HDL-c: high-density lipoprotein cholesterol
ROS: Reactive oxygen species
ATP: adenosine triphosphate
SOD: superoxide dismutase
DIA: data-independent acquisition
LC-MS/MS: liquid chromatography-tandem mass spectrometry
SDS‒PAGE: sodium dodecyl sulfate-polyacrylamide gel electrophoresis
HPLC‒MS/MS: nanohigh-performance liquid chromatography‒mass tandem spectrometry
iRT: interactive response technology
FDR: false discovery rate
DEPs: differentially expressed proteins
PCA: principal component analysis
ITGA2B: integrin subunit alpha 2b
VCL: vinculin
CFD: complement factor D
FGA: fibrinogen alpha chain
VCAM1: vascular cell adhesion molecule 1
CFHR2: complement factor H related 2
VCL: vinculin
LBP: lipopolysaccharide-binding protein
AHSG: alpha 2-HS glycoprotein
IGF2: insulin-like growth Factor 2
IGFALS: insulin-like growth factor binding protein acid labile subunit
IGFBP3: insulin-like growth factor binding protein 3
APOL1: apolipoprotein L1
BCHE: butyrylcholinesterase
CNDP1: carnosine dipeptidase 1
APOA2: apolipoprotein A2
GPLD1: glycosylphosphatidylinositol specific phospholipase D1
LCAT: lecithin-cholesterol acyltransferase
