# Supplementary material for: Exploring the Plasma Proteome: Identifying Hub Proteins linking Aging, Homeostasis, and Organ Function
Source: Int J Med Sci. 2025 Feb 10;22(5):1109–23. doi: 10.7150/ijms.107750 (PMC11866526; doi:10.7150/ijms.107750)

Supplemental Material

Supplemental Tables

Supplemental Table S1. The final proteins after removing missing values across samples.

Protein	DIA_Qvalue	Description
A0A3Q1LSF0	0.003	-
A0A3Q1M3L6	2.166E-04	-
A0A3Q1NGH3	0.003	-
A1BG	0.001	alpha-1-B glycoprotein
A1BG_1	2.546E-07	alpha-1-B glycoprotein
A2M	1.624E-05	alpha-2-macroglobulin
ABCC4	0.003	ATP binding cassette subfamily C member 4
ABI3BP	2.576E-04	ABI family member 3 binding protein
ABRACL	0.003	ABRA C-terminal like
AC002094.3	0.003	novel protein, readthrough between VTN and SEBOX
AC104389.6	0.003	novel protein
AC135068.9	0.002	novel gene identicle to IGHV1OR15-1
ACTA1	0.003	actin alpha 1, skeletal muscle
ACTB	0.003	actin beta
ACTBL2	1.767E-07	actin beta like 2
ACTC1	0.003	actin alpha cardiac muscle 1
ACTG1	9.782E-04	actin gamma 1
ACTN1	0.003	actinin alpha 1
ACTR3	0.003	actin related protein 3
ADAMTS13	0.003	ADAM metallopeptidase with thrombospondin type 1 motif 13
ADAMTSL4	0.004	ADAMTS like 4
ADARB1	0.003	adenosine deaminase RNA specific B1
ADGRF5	0.005	adhesion G protein-coupled receptor F5
ADIPOQ	0.003	adiponectin, C1Q and collagen domain containing
AFM	4.470E-04	afamin
AGT	0.001	angiotensinogen
AHSG	9.185E-05	alpha 2-HS glycoprotein
AL049629.2	0.003	novel protein
AL049634.2	0.003	novel protein, SIRPB1-SIRPD readthrough
AL645922.1	4.470E-04	novel complement component 2 (C2) and complement factor B (CFB) protein
AL807752.7	0.003	novel transcript
ALAD	0.003	aminolevulinate dehydratase
ALB	1.767E-07	albumin
ALB_1	0.003	albumin
ALB_2	0.003	albumin
ALDOA	0.003	aldolase, fructose-bisphosphate A
ALDOB	0.003	aldolase, fructose-bisphosphate B

AMBP	0.002	alpha-1-microglobulin/bikunin precursor
ANG	0.003	angiogenin
ANGPTL3	0.003	angiopoietin like 3
ANPEP	0.003	alanyl aminopeptidase, membrane
ANXA1	0.003	annexin A1
ANXA3	0.003	annexin A3
ANXA6	0.003	annexin A6
AOC3	0.004	amine oxidase copper containing 3
AP2A1	0.004	adaptor related protein complex 2 subunit alpha 1
AP2A2	0.004	adaptor related protein complex 2 subunit alpha 2
APCS	0.003	amyloid P component, serum
APEH	0.004	acylaminoacyl-peptide hydrolase
APMAP	0.003	adipocyte plasma membrane associated protein
APOA1	3.789E-07	apolipoprotein A1
APOA2	1.767E-07	apolipoprotein A2
APOA4	0.001	apolipoprotein A4
APOA5	0.008	apolipoprotein A5
APOB	2.540E-07	apolipoprotein B
APOC1	6.648E-07	apolipoprotein C1
APOC3	0.001	apolipoprotein C3
APOC4	0.003	apolipoprotein C4
APOC4-APOC2	0.003	APOC4-APOC2 readthrough (NMD candidate)
APOC4-APOC2_1	0.002	APOC4-APOC2 readthrough (NMD candidate)
APOD	0.001	apolipoprotein D
APOE	0.002	apolipoprotein E
APOF	0.001	apolipoprotein F
APOH	1.971E-04	apolipoprotein H
APOL1	1.767E-07	apolipoprotein L1
APOL3	0.003	apolipoprotein L3
APOM	0.003	apolipoprotein M
APOM_1	1.944E-04	apolipoprotein M
ARF1	0.003	ADP ribosylation factor 1
ARF3	0.003	ADP ribosylation factor 3
ARHGDIB	0.003	Rho GDP dissociation inhibitor beta
ARPC1B	0.004	actin related protein 2/3 complex subunit 1B
ASGR2	0.004	asialoglycoprotein receptor 2
ATRN	0.002	attractin
AZGP1	1.147E-04	alpha-2-glycoprotein 1, zinc-binding
B2M	0.003	beta-2-microglobulin
B3GNT2	0.003	UDP-GlcNAc:betaGal beta-1,3-N-acetylglucosaminyltransferase 2
BCHE	0.002	butyrylcholinesterase
BLVRB	0.003	biliverdin reductase B
BNC2	0.003	basonuclin 2

BPGM	0.003	bisphosphoglycerate mutase
BTD	0.003	biotinidase
C18orf63	0.003	chromosome 18 open reading frame 63
C1QA	3.866E-04	complement C1q A chain
C1QB	0.003	complement C1q B chain
C1QC	0.003	complement C1q C chain
C1R	0.001	complement C1r
C1RL	0.003	complement C1r subcomponent like
C1S	0.003	complement C1s
C1S_1	0.003	complement C1s
C2	0.003	complement C2
C2_1	0.003	complement C2
C3	1.767E-07	complement C3
C3_1	0.003	complement C3
C3_2	0.003	complement C3
C4A	4.021E-07	complement C4A (Rodgers blood group)
C4B	6.751E-05	complement C4B (Chido blood group)
C4BPA	5.266E-04	complement component 4 binding protein alpha
C4BPB	2.864E-04	complement component 4 binding protein beta
C5	1.939E-04	complement C5
C6	0.001	complement C6
C7	3.562E-04	complement C7
C8A	0.003	complement C8 alpha chain
C8B	8.263E-04	complement C8 beta chain
C8G	7.890E-04	complement C8 gamma chain
C9	9.155E-04	complement C9
CA1	0.003	carbonic anhydrase 1
CA2	0.003	carbonic anhydrase 2
CALM1	0.003	calmodulin 1
CALM2	0.003	calmodulin 2
CALM3	0.003	calmodulin 3
CALR	0.003	calreticulin
CAMP	0.003	cathelicidin antimicrobial peptide
CAP1	0.003	cyclase associated actin cytoskeleton regulatory protein 1
CAPZA2	0.003	capping actin protein of muscle Z-line subunit alpha 2
CAT	0.003	catalase
CAVIN2	0.003	caveolae associated protein 2
CD109	0.003	CD109 molecule
CD14	0.003	CD14 molecule
CD163	0.003	CD163 molecule
CD44	0.003	CD44 molecule (Indian blood group)
CD59	0.003	CD59 molecule (CD59 blood group)
CD5L	0.002	CD5 molecule like
CD93	0.004	CD93 molecule

CDC42	0.003	cell division cycle 42
CDC5L	1.059E-04	cell division cycle 5 like
CDH1	0.003	cadherin 1
CDH13	0.003	cadherin 13
CDH5	3.773E-04	cadherin 5
CETP	0.003	cholesteryl ester transfer protein
CFD	0.003	complement factor D
CFH	9.398E-04	complement factor H
CFHR1	0.002	complement factor H related 1
CFHR2	0.003	complement factor H related 2
CFHR3	1.992E-04	complement factor H related 3
CFHR4	0.003	complement factor H related 4
CFHR5	0.003	complement factor H related 5
CFI	2.051E-04	complement factor I
CFL1	0.003	cofilin 1
CFP	0.003	complement factor properdin
CHL1	0.003	cell adhesion molecule L1 like
CLC	0.003	Charcot-Leyden crystal galectin
CLEC3B	0.003	C-type lectin domain family 3 member B
CLIC1	0.003	chloride intracellular channel 1
CLU	2.452E-04	clusterin
CNDP1	3.892E-04	carnosine dipeptidase 1
CNN2	0.003	calponin 2
CNTN1	0.003	contactin 1
COL18A1	0.004	collagen type XVIII alpha 1 chain
COL1A1	0.003	collagen type I alpha 1 chain
COL6A1	0.003	collagen type VI alpha 1 chain
COL6A3	0.003	collagen type VI alpha 3 chain
COLEC10	0.003	collectin subfamily member 10
COLEC11	0.003	collectin subfamily member 11
COMP	0.003	cartilage oligomeric matrix protein
CORO1A	0.001	coronin 1A
COTL1	0.003	coactosin like F-actin binding protein 1
CP	2.335E-07	ceruloplasmin
CP_1	0.011	ceruloplasmin
CPB2	0.003	carboxypeptidase B2
CPN1	0.003	carboxypeptidase N subunit 1
CPN2	7.221E-04	carboxypeptidase N subunit 2
CRISP3	0.003	cysteine rich secretory protein 3
CRP	0.002	C-reactive protein
CRTAC1	0.003	cartilage acidic protein 1
CSF1R	0.003	colony stimulating factor 1 receptor
CST3	0.003	cystatin C
CTBS	0.003	chitobiase

CTSB	0.008	cathepsin B
CTSD	0.003	cathepsin D
CTSG	0.003	cathepsin G
DAG1	0.005	dystroglycan 1
DBH	0.003	dopamine beta-hydroxylase
DDT	0.003	D-dopachrome tautomerase
DEFA1	0.003	defensin alpha 1
DEFA1B	0.003	defensin alpha 1B
DEFA3	0.003	defensin alpha 3
DPT	0.004	dermatopontin
E1BNR0	0.003	-
ECM1	0.003	extracellular matrix protein 1
EEF1A1	0.003	eukaryotic translation elongation factor 1 alpha 1
EEF1A2	0.003	eukaryotic translation elongation factor 1 alpha 2
EFEMP1	0.003	EGF containing fibulin extracellular matrix protein 1
EFEMP2	0.003	EGF containing fibulin extracellular matrix protein 2
EIF3G	0.003	eukaryotic translation initiation factor 3 subunit G
EIF5A	0.004	eukaryotic translation initiation factor 5A
EIF5A2	0.004	eukaryotic translation initiation factor 5A2
ELP3	5.036E-04	elongator acetyltransferase complex subunit 3
ENO1	0.003	enolase 1
ENPP2	0.003	ectonucleotide pyrophosphatase/phosphodiesterase 2
ERN1	1.834E-05	endoplasmic reticulum to nucleus signaling 1
ESD	0.005	esterase D
F10	0.003	coagulation factor X
F11	0.001	coagulation factor XI
F11R	0.003	F11 receptor
F12	0.002	coagulation factor XII
F13A1	9.185E-05	coagulation factor XIII A chain
F13B	0.002	coagulation factor XIII B chain
F1MVK1	0.002	-
F1N076	0.003	-
F2	4.278E-04	coagulation factor II, thrombin
F5	0.003	coagulation factor V
F7	0.003	coagulation factor VII
F9	0.003	coagulation factor IX
FAH	0.003	fumarylacetoacetate hydrolase
FBLN1	0.002	fibulin 1
FBLN1_1	0.003	fibulin 1
FBLN5	0.004	fibulin 5
FCGBP	0.003	Fc fragment of IgG binding protein
FCGR2A	0.005	Fc fragment of IgG receptor IIa
FCGR3A	0.003	Fc fragment of IgG receptor IIIa
FCN2	0.003	ficolin 2

FCN3	0.003	ficolin 3
FERMT3	0.003	fermitin family member 3
FETUB	0.003	fetuin B
FGA	1.767E-07	fibrinogen alpha chain
FGB	7.470E-06	fibrinogen beta chain
FGFBP2	0.004	fibroblast growth factor binding protein 2
FGG	1.413E-05	fibrinogen gamma chain
FGG_1	0.003	fibrinogen gamma chain
FGL1	0.003	fibrinogen like 1
FKBP1A	0.003	FKBP prolyl isomerase 1A
FLNA	0.003	filamin A
FN1	1.738E-05	fibronectin 1
FN1_1	0.003	fibronectin 1
FN1_2	0.003	fibronectin 1
FP565260.3	0.003	ICOS ligand
FTO	0.002	FTO alpha-ketoglutarate dependent dioxygenase
FUCA1	0.003	alpha-L-fucosidase 1
G3MYZ3	1.767E-07	-
G6PD	0.003	glucose-6-phosphate dehydrogenase
GAPDH	0.001	glyceraldehyde-3-phosphate dehydrogenase
GC	1.168E-04	GC vitamin D binding protein
GCA	0.003	grancalcin
GDI2	0.003	GDP dissociation inhibitor 2
GGH	0.003	gamma-glutamyl hydrolase
GLIPR2	0.006	GLI pathogenesis related 2
GMPPB	0.003	GDP-mannose pyrophosphorylase B
GNPTG	0.004	N-acetylglucosamine-1-phosphate transferase subunit gamma
GP1BA	0.003	glycoprotein Ib platelet subunit alpha
GP5	0.003	glycoprotein V platelet
GPI	0.003	glucose-6-phosphate isomerase
GPLD1	0.002	glycosylphosphatidylinositol specific phospholipase D1
GPX3	0.003	glutathione peroxidase 3
GSN	7.338E-04	gelsolin
GSTO1	0.003	glutathione S-transferase omega 1
GSTP1	0.003	glutathione S-transferase pi 1
H4-16	0.003	H4 histone 16
H4C1	0.003	H4 clustered histone 1
H4C11	0.003	H4 clustered histone 11
H4C12	0.003	H4 clustered histone 12
H4C13	0.003	H4 clustered histone 13
H4C14	0.003	H4 clustered histone 14
H4C15	0.003	H4 clustered histone 15
H4C2	0.003	H4 clustered histone 2
H4C3	0.003	H4 clustered histone 3

H4C4	0.003	H4 clustered histone 4
H4C5	0.003	H4 clustered histone 5
H4C6	0.003	H4 clustered histone 6
H4C8	0.003	H4 clustered histone 8
H4C9	0.003	H4 clustered histone 9
HABP2	0.003	hyaluronan binding protein 2
HBA1	1.319E-04	hemoglobin subunit alpha 1
HBA1_1	0.003	hemoglobin subunit alpha 1
HBA2	1.319E-04	hemoglobin subunit alpha 2
HBA2_1	0.003	hemoglobin subunit alpha 2
HBB	2.483E-05	hemoglobin subunit beta
HBD	0.003	hemoglobin subunit delta
HBE1	0.003	hemoglobin subunit epsilon 1
HGFAC	0.003	HGF activator
HK3	0.004	hexokinase 3
HLA-B	0.003	major histocompatibility complex, class I, B
HP	1.767E-07	haptoglobin
HPN	0.005	hepsin
HPR	0.003	haptoglobin-related protein
HPX	2.582E-06	hemopexin
HRG	5.528E-04	histidine rich glycoprotein
HSP90AA1	0.003	heat shock protein 90 alpha family class A member 1
HSP90B1	0.003	heat shock protein 90 beta family member 1
HSPA1A	0.003	heat shock protein family A (Hsp70) member 1A
HSPA1B	0.003	heat shock protein family A (Hsp70) member 1B
HSPA5	0.003	heat shock protein family A (Hsp70) member 5
HSPA8	0.003	heat shock protein family A (Hsp70) member 8
HSPG2	1.767E-07	heparan sulfate proteoglycan 2
HYOU1	0.004	hypoxia up-regulated 1
ICAM1	0.003	intercellular adhesion molecule 1
ICAM2	0.003	intercellular adhesion molecule 2
ICOSLG	0.003	inducible T cell costimulator ligand
IGF1	0.003	insulin like growth factor 1
IGF2	0.003	insulin like growth factor 2
IGFALS	0.001	insulin like growth factor binding protein acid labile subunit
IGFBP2	0.003	insulin like growth factor binding protein 2
IGFBP3	0.003	insulin like growth factor binding protein 3
IGFBP4	0.003	insulin like growth factor binding protein 4
IGFBP5	0.004	insulin like growth factor binding protein 5
IGFBP6	0.004	insulin like growth factor binding protein 6
IGFBP7	0.005	insulin like growth factor binding protein 7
IGHA1	5.952E-06	immunoglobulin heavy constant alpha 1
IGHA2	7.705E-06	immunoglobulin heavy constant alpha 2 (A2m marker)
IGHD	0.003	immunoglobulin heavy constant delta

IGHG1	1.767E-07	immunoglobulin heavy constant gamma 1 (G1m marker)
IGHG2	1.767E-07	immunoglobulin heavy constant gamma 2 (G2m marker)
IGHG3	1.076E-04	immunoglobulin heavy constant gamma 3 (G3m marker)
IGHG4	3.653E-05	immunoglobulin heavy constant gamma 4 (G4m marker)
IGHM	3.623E-07	immunoglobulin heavy constant mu
IGHV1-18	0.003	immunoglobulin heavy variable 1-18
IGHV1-2	1.046E-05	immunoglobulin heavy variable 1-2
IGHV1-24	0.003	immunoglobulin heavy variable 1-24
IGHV1-3	0.003	immunoglobulin heavy variable 1-3
IGHV1-45	0.003	immunoglobulin heavy variable 1-45
IGHV1-46	0.003	immunoglobulin heavy variable 1-46
IGHV1-69	0.002	immunoglobulin heavy variable 1-69
IGHV1-69-2	0.003	immunoglobulin heavy variable 1-69-2
IGHV1-69D	0.003	immunoglobulin heavy variable 1-69D
IGHV1OR15-1	0.002	immunoglobulin heavy variable 1/OR15-1 (non-functional)
IGHV1OR15-9	0.003	immunoglobulin heavy variable 1/OR15-9 (non-functional)
IGHV1OR21-1	0.003	immunoglobulin heavy variable 1/OR21-1 (non-functional)
IGHV2-26	4.021E-07	immunoglobulin heavy variable 2-26
IGHV2-5	0.003	immunoglobulin heavy variable 2-5
IGHV2-70	0.003	immunoglobulin heavy variable 2-70
IGHV2-70D	0.003	immunoglobulin heavy variable 2-70D
IGHV3-11	0.003	immunoglobulin heavy variable 3-11
IGHV3-13	0.003	immunoglobulin heavy variable 3-13
IGHV3-15	0.003	immunoglobulin heavy variable 3-15
IGHV3-16	0.008	immunoglobulin heavy variable 3-16 (non-functional)
IGHV3-20	0.003	immunoglobulin heavy variable 3-20
IGHV3-21	0.003	immunoglobulin heavy variable 3-21
IGHV3-23	0.003	immunoglobulin heavy variable 3-23
IGHV3-30	1.135E-05	immunoglobulin heavy variable 3-30
IGHV3-33	0.003	immunoglobulin heavy variable 3-33
IGHV3-35	0.003	immunoglobulin heavy variable 3-35 (non-functional)
IGHV3-38	0.003	immunoglobulin heavy variable 3-38 (non-functional)
IGHV3-43	0.002	immunoglobulin heavy variable 3-43
IGHV3-48	0.003	immunoglobulin heavy variable 3-48
IGHV3-49	0.002	immunoglobulin heavy variable 3-49
IGHV3-53	2.197E-04	immunoglobulin heavy variable 3-53
IGHV3-64	8.552E-05	immunoglobulin heavy variable 3-64
IGHV3-64D	1.461E-04	immunoglobulin heavy variable 3-64D
IGHV3-7	8.263E-04	immunoglobulin heavy variable 3-7
IGHV3-72	5.000E-04	immunoglobulin heavy variable 3-72
IGHV3-73	0.003	immunoglobulin heavy variable 3-73
IGHV3-74	0.003	immunoglobulin heavy variable 3-74
IGHV3OR15-7	0.003	immunoglobulin heavy variable 3/OR15-7 (pseudogene)
IGHV3OR16-10	0.004	immunoglobulin heavy variable 3/OR16-10 (non-functional)

IGHV3OR16-12	0.003	immunoglobulin heavy variable 3/OR16-12 (non-functional)
IGHV3OR16-13	0.001	immunoglobulin heavy variable 3/OR16-13 (non-functional)
IGHV3OR16-9	0.002	immunoglobulin heavy variable 3/OR16-9 (non-functional)
IGHV4-28	0.003	immunoglobulin heavy variable 4-28
IGHV4-31	0.003	immunoglobulin heavy variable 4-31
IGHV4-34	0.001	immunoglobulin heavy variable 4-34
IGHV4-39	0.003	immunoglobulin heavy variable 4-39
IGHV4-4	0.003	immunoglobulin heavy variable 4-4
IGHV4-59	0.003	immunoglobulin heavy variable 4-59
IGHV4-61	0.003	immunoglobulin heavy variable 4-61
IGHV4OR15-8	0.003	immunoglobulin heavy variable 4/OR15-8 (non-functional)
IGHV5-10-1	5.266E-04	immunoglobulin heavy variable 5-10-1
IGHV5-51	0.003	immunoglobulin heavy variable 5-51
IGHV6-1	4.613E-04	immunoglobulin heavy variable 6-1
IGHV7-4-1	0.003	immunoglobulin heavy variable 7-4-1
IGHV7-81	0.004	immunoglobulin heavy variable 7-81 (non-functional)
IGKC	1.930E-06	immunoglobulin kappa constant
IGKJ1	0.003	immunoglobulin kappa joining 1
IGKJ3	0.003	immunoglobulin kappa joining 3
IGKV1-12	5.036E-04	immunoglobulin kappa variable 1-12
IGKV1-16	0.003	immunoglobulin kappa variable 1-16
IGKV1-17	0.003	immunoglobulin kappa variable 1-17
IGKV1-27	0.003	immunoglobulin kappa variable 1-27
IGKV1-33	0.002	immunoglobulin kappa variable 1-33
IGKV1-37	0.003	immunoglobulin kappa variable 1-37 (non-functional)
IGKV1-39	0.003	immunoglobulin kappa variable 1-39
IGKV1-5	0.003	immunoglobulin kappa variable 1-5
IGKV1-8	3.190E-04	immunoglobulin kappa variable 1-8
IGKV1D-12	5.036E-04	immunoglobulin kappa variable 1D-12
IGKV1D-13	0.003	immunoglobulin kappa variable 1D-13
IGKV1D-16	7.660E-04	immunoglobulin kappa variable 1D-16
IGKV1D-17	0.004	immunoglobulin kappa variable 1D-17
IGKV1D-33	0.002	immunoglobulin kappa variable 1D-33
IGKV1D-37	0.003	immunoglobulin kappa variable 1D-37 (non-functional)
IGKV1D-39	0.003	immunoglobulin kappa variable 1D-39
IGKV1D-8	0.004	immunoglobulin kappa variable 1D-8
IGKV2-24	0.003	immunoglobulin kappa variable 2-24
IGKV2-30	1.662E-04	immunoglobulin kappa variable 2-30
IGKV2-40	0.001	immunoglobulin kappa variable 2-40
IGKV2D-24	0.003	immunoglobulin kappa variable 2D-24 (non-functional)
IGKV2D-28	0.002	immunoglobulin kappa variable 2D-28
IGKV2D-29	0.002	immunoglobulin kappa variable 2D-29
IGKV2D-40	0.001	immunoglobulin kappa variable 2D-40
IGKV3-11	1.992E-04	immunoglobulin kappa variable 3-11

IGKV3-15	1.939E-04	immunoglobulin kappa variable 3-15
IGKV3-20	1.767E-07	immunoglobulin kappa variable 3-20
IGKV3-7	0.003	immunoglobulin kappa variable 3-7 (non-functional)
IGKV3D-15	0.003	immunoglobulin kappa variable 3D-15
IGKV3D-20	0.001	immunoglobulin kappa variable 3D-20
IGKV3D-7	9.782E-04	immunoglobulin kappa variable 3D-7
IGKV3OR2-268	9.782E-04	immunoglobulin kappa variable 3/OR2-268 (non-functional)
IGKV4-1	5.744E-04	immunoglobulin kappa variable 4-1
IGKV6-21	0.003	immunoglobulin kappa variable 6-21 (non-functional)
IGKV6D-21	0.003	immunoglobulin kappa variable 6D-21 (non-functional)
IGLC2	1.767E-07	immunoglobulin lambda constant 2
IGLC3	1.767E-07	immunoglobulin lambda constant 3 (Kern-Oz+ marker)
IGLC7	3.321E-05	immunoglobulin lambda constant 7
IGLL1	1.767E-07	immunoglobulin lambda like polypeptide 1
IGLL1_1	0.003	immunoglobulin lambda like polypeptide 1
IGLL5	2.302E-04	immunoglobulin lambda like polypeptide 5
IGLV10-54	0.003	immunoglobulin lambda variable 10-54
IGLV1-36	0.003	immunoglobulin lambda variable 1-36
IGLV1-40	2.582E-06	immunoglobulin lambda variable 1-40
IGLV1-44	0.003	immunoglobulin lambda variable 1-44
IGLV1-47	0.002	immunoglobulin lambda variable 1-47
IGLV1-51	0.003	immunoglobulin lambda variable 1-51
IGLV2-11	0.003	immunoglobulin lambda variable 2-11
IGLV2-14	0.003	immunoglobulin lambda variable 2-14
IGLV2-18	0.003	immunoglobulin lambda variable 2-18
IGLV2-23	0.003	immunoglobulin lambda variable 2-23
IGLV2-33	0.003	immunoglobulin lambda variable 2-33 (non-functional)
IGLV2-8	0.002	immunoglobulin lambda variable 2-8
IGLV3-1	0.003	immunoglobulin lambda variable 3-1
IGLV3-10	0.003	immunoglobulin lambda variable 3-10
IGLV3-19	1.346E-04	immunoglobulin lambda variable 3-19
IGLV3-21	0.002	immunoglobulin lambda variable 3-21
IGLV3-25	0.003	immunoglobulin lambda variable 3-25
IGLV3-27	0.003	immunoglobulin lambda variable 3-27
IGLV3-9	0.003	immunoglobulin lambda variable 3-9
IGLV4-60	0.003	immunoglobulin lambda variable 4-60
IGLV4-69	0.003	immunoglobulin lambda variable 4-69
IGLV5-37	0.003	immunoglobulin lambda variable 5-37
IGLV5-45	0.002	immunoglobulin lambda variable 5-45
IGLV5-48	0.003	immunoglobulin lambda variable 5-48 (non-functional)
IGLV5-52	0.003	immunoglobulin lambda variable 5-52
IGLV6-57	0.003	immunoglobulin lambda variable 6-57
IGLV7-43	0.003	immunoglobulin lambda variable 7-43
IGLV8-61	0.002	immunoglobulin lambda variable 8-61

IGLV9-49	0.003	immunoglobulin lambda variable 9-49
IL12RB2	0.003	interleukin 12 receptor subunit beta 2
IL1RAP	0.003	interleukin 1 receptor accessory protein
INHBC	0.003	inhibin subunit beta C
IPMK	0.003	inositol polyphosphate multikinase
iRT-Kit_WR_fusion	5.266E-04	-
ITGA2B	0.003	integrin subunit alpha 2b
ITGB3	0.003	integrin subunit beta 3
ITIH1	2.181E-04	inter-alpha-trypsin inhibitor heavy chain 1
ITIH2	6.568E-06	inter-alpha-trypsin inhibitor heavy chain 2
ITIH3	6.740E-04	inter-alpha-trypsin inhibitor heavy chain 3
ITIH4	1.059E-04	inter-alpha-trypsin inhibitor heavy chain 4
ITIH4_1	0.003	inter-alpha-trypsin inhibitor heavy chain 4
ITLN1	0.003	intelectin 1
JCHAIN	0.002	joining chain of multimeric IgA and IgM
KIAA0100	0.003	KIAA0100
KIAA1958	0.003	KIAA1958
KLKB1	6.194E-05	kallikrein B1
KNG1	6.080E-05	kininogen 1
KNG1_1	0.003	kininogen 1
KRT1	0.003	keratin 1
KRT10	0.003	keratin 10
KRT15	0.003	keratin 15
KRT16	0.003	keratin 16
KRT2	0.003	keratin 2
KRT31	0.003	keratin 31
KRT33B	0.003	keratin 33B
KRT34	0.003	keratin 34
KRT6A	0.003	keratin 6A
KRT85	0.003	keratin 85
KRT9	0.003	keratin 9
KRTAP2-1	0.003	keratin associated protein 2-1
KRTAP2-2	0.003	keratin associated protein 2-2
KRTAP2-3	0.003	keratin associated protein 2-3
KRTAP2-4	0.003	keratin associated protein 2-4
KRTDAP	0.003	keratinocyte differentiation associated protein
LBP	0.003	lipopolysaccharide binding protein
LCAT	0.003	lecithin-cholesterol acyltransferase
LCN2	0.004	lipocalin 2
LCP1	0.003	lymphocyte cytosolic protein 1
LDHA	0.003	lactate dehydrogenase A
LDHB	0.003	lactate dehydrogenase B
LGALS1	0.003	galectin 1
LGALS3BP	0.003	galectin 3 binding protein

LPA	0.003	lipoprotein(a)
LRG1	0.001	leucine rich alpha-2-glycoprotein 1
LRP1	0.006	LDL receptor related protein 1
LTA4H	0.003	leukotriene A4 hydrolase
LTBP1	0.004	latent transforming growth factor beta binding protein 1
LTF	0.003	lactotransferrin
LUM	0.003	lumican
LYVE1	0.003	lymphatic vessel endothelial hyaluronan receptor 1
LYZ	0.003	lysozyme
MAN1A1	0.003	mannosidase alpha class 1A member 1
MAN2A1	0.005	mannosidase alpha class 2A member 1
MASP1	0.003	mannan binding lectin serine peptidase 1
MASP1_1	3.773E-04	mannan binding lectin serine peptidase 1
MASP2	0.003	mannan binding lectin serine peptidase 2
MBL2	0.003	mannose binding lectin 2
MCAM	0.003	melanoma cell adhesion molecule
MEGF8	0.008	multiple EGF like domains 8
MGP	0.004	matrix Gla protein
MIF	0.003	macrophage migration inhibitory factor
MINPP1	0.003	multiple inositol-polyphosphate phosphatase 1
MMP16	0.003	matrix metallopeptidase 16
MMP2	6.245E-06	matrix metallopeptidase 2
MMRN1	0.004	multimerin 1
MMRN2	0.003	multimerin 2
MRC1	0.003	mannose receptor C-type 1
MSN	0.003	moesin
MST1	0.003	macrophage stimulating 1
MUC16	0.003	mucin 16, cell surface associated
MYH9	0.003	myosin heavy chain 9
NCAM1	0.003	neural cell adhesion molecule 1
NDUFA5	0.003	NADH:ubiquinone oxidoreductase subunit A5
NID1	0.003	nidogen 1
NME1-NME2	0.003	NME1-NME2 readthrough
NME2	0.003	NME/NM23 nucleoside diphosphate kinase 2
NPC2	0.008	NPC intracellular cholesterol transporter 2
NRP1	0.003	neuropilin 1
NSD2	0.006	nuclear receptor binding SET domain protein 2
OAF	0.003	out at first homolog
OIT3	0.004	oncoprotein induced transcript 3
OLFM1	0.003	olfactomedin 1
OMD	0.004	osteomodulin
ORM1	2.181E-06	orosomucoid 1
ORM2	1.939E-04	orosomucoid 2
OSTF1	0.003	osteoclast stimulating factor 1

P01044	1.971E-04	-
P01045	1.971E-04	-
P02768	1.767E-07	-
PARK7	0.003	Parkinsonism associated deglycase
PCDHGC5	0.004	protocadherin gamma subfamily C, 5
PCOLCE	0.003	procollagen C-endopeptidase enhancer
PCSK9	0.003	proprotein convertase subtilisin/kexin type 9
PCYOX1	0.003	prenylcysteine oxidase 1
PDIA3	0.003	protein disulfide isomerase family A member 3
PDLIM1	0.003	PDZ and LIM domain 1
PEBP1	0.005	phosphatidylethanolamine binding protein 1
PEPD	0.003	peptidase D
PF4	0.003	platelet factor 4
PF4V1	0.001	platelet factor 4 variant 1
PFN1	0.003	profilin 1
PGAM1	0.003	phosphoglycerate mutase 1
PGD	0.003	phosphogluconate dehydrogenase
PGK1	0.003	phosphoglycerate kinase 1
PGLYRP2	0.002	peptidoglycan recognition protein 2
PI16	0.003	peptidase inhibitor 16
PIGR	0.003	polymeric immunoglobulin receptor
PKM	0.003	pyruvate kinase M1/2
PLA2G7	0.003	phospholipase A2 group VII
PLG	1.662E-04	plasminogen
PLTP	6.194E-05	phospholipid transfer protein
PLXDC2	0.003	plexin domain containing 2
PNP	0.003	purine nucleoside phosphorylase
PON1	0.003	paraoxonase 1
PON3	0.003	paraoxonase 3
POSTN	0.003	periostin
PPBP	0.003	pro-platelet basic protein
PPIA	0.003	peptidylprolyl isomerase A
PRDX1	0.002	peroxiredoxin 1
PRDX2	0.003	peroxiredoxin 2
PRDX5	0.003	peroxiredoxin 5
PRDX6	0.003	peroxiredoxin 6
PRG2	0.003	proteoglycan 2, pro eosinophil major basic protein
PRG4	2.302E-04	proteoglycan 4
PROC	0.003	protein C, inactivator of coagulation factors Va and VIIIa
PROCR	0.003	protein C receptor
PROS1	1.939E-04	protein S
PROZ	0.003	protein Z, vitamin K dependent plasma glycoprotein
PRSS1	0.003	serine protease 1
PRSS3	0.003	serine protease 3

PRTN3	0.003	proteinase 3
PTGDS	0.003	prostaglandin D2 synthase
PTMA	0.007	prothymosin alpha
PTPRG	0.004	protein tyrosine phosphatase receptor type G
PTPRJ	0.004	protein tyrosine phosphatase receptor type J
PVR	0.003	PVR cell adhesion molecule
PZP	0.003	PZP alpha-2-macroglobulin like
QSOX1	0.003	quiescin sulfhydryl oxidase 1
RABGEF1	0.003	RAB guanine nucleotide exchange factor 1
RAC1	0.003	Rac family small GTPase 1
RAC2	0.004	Rac family small GTPase 2
RAN	0.004	RAN, member RAS oncogene family
RAP1B	0.003	RAP1B, member of RAS oncogene family
RARRES2	0.003	retinoic acid receptor responder 2
RBP4	0.002	retinol binding protein 4
REG3A	0.003	regenerating family member 3 alpha
RHOA	0.003	ras homolog family member A
RMDN2	0.004	regulator of microtubule dynamics 2
RNASE1	0.003	ribonuclease A family member 1, pancreatic
RNASE4	0.003	ribonuclease A family member 4
RNH1	0.003	ribonuclease/angiogenin inhibitor 1
S100A11	0.003	S100 calcium binding protein A11
S100A12	0.003	S100 calcium binding protein A12
S100A4	0.003	S100 calcium binding protein A4
S100A6	0.003	S100 calcium binding protein A6
S100A8	2.546E-07	S100 calcium binding protein A8
S100A9	8.873E-04	S100 calcium binding protein A9
SAA1	0.001	serum amyloid A1
SAA2	2.046E-04	serum amyloid A2
SAA4	5.447E-04	serum amyloid A4, constitutive
SELENBP1	0.003	selenium binding protein 1
SELL	0.003	selectin L
SEMA4B	0.004	semaphorin 4B
SERPINA1	4.624E-07	serpin family A member 1
SERPINA10	0.003	serpin family A member 10
SERPINA11	0.004	serpin family A member 11
SERPINA3	1.971E-04	serpin family A member 3
SERPINA3_1	0.002	serpin family A member 3
SERPINA4	7.338E-04	serpin family A member 4
SERPINA5	0.003	serpin family A member 5
SERPINA6	3.838E-06	serpin family A member 6
SERPINA7	0.002	serpin family A member 7
SERPINB1	0.003	serpin family B member 1
SERPINC1	8.263E-04	serpin family C member 1

SERPIND1	0.001	serpin family D member 1
SERPINF1	0.003	serpin family F member 1
SERPINF2	1.767E-07	serpin family F member 2
SERPING1	9.045E-06	serpin family G member 1
SH3BGRL3	0.004	SH3 domain binding glutamate rich protein like 3
SHBG	0.003	sex hormone binding globulin
SIRPA	0.003	signal regulatory protein alpha
SIRPB1	0.003	signal regulatory protein beta 1
SIRPG	0.003	signal regulatory protein gamma
SLC4A1	0.003	solute carrier family 4 member 1 (Diego blood group)
SOD1	0.004	superoxide dismutase 1
SOD3	0.003	superoxide dismutase 3
SP100	0.005	SP100 nuclear antigen
SPARC	0.003	secreted protein acidic and cysteine rich
SPARCL1	0.004	SPARC like 1
SPP2	0.003	secreted phosphoprotein 2
SRGN	0.004	serglycin
SUN1	0.003	Sad1 and UNC84 domain containing 1
TAGLN2	0.003	transgelin 2
TALDO1	0.003	transaldolase 1
TF	1.767E-07	transferrin
TF_1	0.003	transferrin
TF_2	0.003	transferrin
TFRC	0.003	transferrin receptor
TGFBI	1.309E-04	transforming growth factor beta induced
THBS1	0.002	thrombospondin 1
THBS4	0.003	thrombospondin 4
TIMP1	0.003	TIMP metallopeptidase inhibitor 1
TIMP2	0.007	TIMP metallopeptidase inhibitor 2
TKT	0.003	transketolase
TLN1	0.003	talin 1
TMC8	0.003	transmembrane channel like 8
TMSB10	0.003	thymosin beta 10
TMSB4X	0.002	thymosin beta 4 X-linked
TNC	0.004	tenascin C
TNXB	0.003	tenascin XB
TPI1	0.003	triosephosphate isomerase 1
TPM3	0.003	tropomyosin 3
TPM4	0.003	tropomyosin 4
TTR	0.002	transthyretin
TUBA1B	0.003	tubulin alpha 1b
TUBB	0.003	tubulin beta class I
TXN	0.003	thioredoxin
USP19	0.005	ubiquitin specific peptidase 19

VASN	0.003	vasorin
VCAM1	0.003	vascular cell adhesion molecule 1
VCL	0.003	vinculin
VIM	0.003	vimentin
VNN1	0.003	vanin 1
VPS13A	0.003	vacuolar protein sorting 13 homolog A
VTN	1.767E-07	vitronectin
VWF	0.002	von Willebrand factor
WDR1	0.003	WD repeat domain 1
WRAP73	0.003	WD repeat containing, antisense to TP73
YWHAB	0.003	tyrosine 3-monooxygenase/tryptophan 5-monooxygenase activation protein beta
YWHAE	0.003	tyrosine 3-monooxygenase/tryptophan 5-monooxygenase activation protein epsilon
YWHAZ	0.003	tyrosine 3-monooxygenase/tryptophan 5-monooxygenase activation protein zeta
ZYX	0.003	zyxin

Supplemental Table S2. Protein correlations with physiological indices.

Index	Protein	EffectSize	AveExpr	t	P.Value	adj.P.Val	B	Sig	Description	KEGG_A_class	KEGG_B_class
SBP	APOL3	-0.121	17.957	-4.926	9.610E-06	0.006	3.116	Down	apolipoprotein L3	-	-
	**BCHE**	0.016	20.144	5.549	1.089E-06	4.159E-04	4.614	Up	butyrylcholinesterase	-	-
	**ITIH3**	-0.014	21.383	-5.511	1.249E-06	4.159E-04	4.479	Down	inter-alpha-trypsin inhibitor heavy chain 3	-	-
	**IGF2**	0.013	20.250	4.562	3.308E-05	5.508E-03	1.264	Up	insulin like growth factor 2	Environmental Information Processing; Human Diseases	Signal transduction; Cancers
	**FETUB**	0.012	20.316	4.444	4.909E-05	0.006	0.880	Up	fetuin B	-	-
	**LRG1**	-0.016	22.436	-4.431	5.135E-05	0.006	0.836	Down	leucine rich alpha-2-glycoprotein 1	-	-
	**AHSG**	0.011	26.205	4.366	6.368E-05	0.006	0.627	Up	alpha 2-HS glycoprotein	-	-
SOD	**IGFALS**	0.020	20.478	4.305	7.786E-05	0.006	0.432	Up	insulin like growth factor binding protein acid labile subunit	Organismal Systems	Endocrine system
	SERPINA4	0.012	21.224	4.270	8.728E-05	0.006	0.321	Up	serpin family A member 4	-	-
	IGLV10.54	0.072	19.666	4.136	1.764E-04	0.010	-0.153	Up	-	-	-
	HSP90B1	-0.097	13.705	-4.013	2.048E-04	0.010	-0.493	Down	heat shock protein 90 beta family member 1	Environmental Information Processing; Human Diseases; Genetic Information Processing; Organismal Systems	Signal transduction; Cancers; Infectious diseases; Folding, sorting and degradation; Cardiovascular diseases; Endocrine system; Immune

											system
	PPBP	-0.021	21.391	-3.996	2.123E-04	0.010	-0.538	Down	pro-platelet basic protein	Organismal Systems; Environmental Information Processing	Immune system; Signaling molecules and interaction
	**ORM1**	-0.013	27.097	-3.986	2.189E-04	0.010	-0.567	Down	orosomucoid 1	-	-
	**EFEMP1**	-0.012	19.259	-3.964	2.350E-04	0.010	-0.636	Down	EGF containing fibulin extracellular matrix protein 1	-	-
	**GPLD1**	0.015	20.225	3.952	2.435E-04	0.010	-0.670	Up	glycosylphosphatidylinositol specific phospholipase D1	Metabolism	Global and overview maps; Glycan biosynthesis and metabolism
	CRP.1	-0.110	18.382	-3.753	4.723E-04	0.017	-1.297	Down	-	-	-
	SERPINA5	0.014	19.675	3.685	5.628E-04	0.017	-1.474	Up	serpin family A member 5	Organismal Systems	Immune system
	**LBP**	-0.016	21.440	-3.670	5.889E-04	0.017	-1.517	Down	lipopolysaccharide binding protein	Human Diseases; Environmental Information Processing; Organismal Systems	Infectious diseases; Signal transduction; Immune system
	**APOA2**	0.010	27.642	3.593	7.462E-04	0.019	-1.743	Up	apolipoprotein A2	Organismal Systems	Endocrine system; Digestive system
	SAA2	-0.049	21.336	-3.576	7.861E-04	0.019	-1.793	Down	serum amyloid A2	-	-
	SAA1	-0.035	22.740	-3.564	8.152E-04	0.019	-1.828	Down	serum amyloid A1	-	-
	C9	-0.010	23.166	-3.563	8.179E-04	0.019	-1.831	Down	complement C9	Human Diseases; Organismal Systems	Immune diseases; Immune system; Neurodegenerative diseases

	**SERPINA3**	-0.010	25.426	-3.537	8.851E-04	0.020	-1.906	Down	serpin family A member 3	-	-
	PON3	0.056	16.050	3.381	0.001	0.028	-2.355	Up	paraoxonase 3	-	-
	VWF	-0.051	16.745	-3.335	0.002	0.030	-2.475	Down	von Willebrand factor	Environmental Information Processing; Cellular Processes; Organismal Systems; Human Diseases	Signal transduction; Cellular community - eukaryotes; Immune system; Signaling molecules and interaction; Infectious diseases
	HPR	0.017	22.683	3.335	0.002	0.030	-2.475	Up	haptoglobin-related protein	Human Diseases	Infectious diseases
	**CFD**	-0.011	19.059	-3.291	0.002	0.033	-2.598	Down	complement factor D	Human Diseases; Organismal Systems	Infectious diseases; Immune system
	PROZ	0.041	17.665	3.152	0.003	0.041	-2.973	Up	protein Z, vitamin K dependent plasma glycoprotein	-	-
Cr	**CFD**	0.023	19.036	4.694	1.345E-05	0.005	2.364	Up	complement factor D	Human Diseases; Organismal Systems	Infectious diseases; Immune system
	**CST3**	0.024	19.665	4.685	1.387E-05	0.005	2.334	Up	cystatin C	Organismal Systems	Digestive system
	PROZ	-0.063	17.866	-4.210	7.662E-05	0.013	0.685	Down	protein Z, vitamin K dependent plasma glycoprotein	-	-
	**EFEMP1**	0.018	19.269	4.210	7.669E-05	0.013	0.684	Up	EGF containing fibulin extracellular matrix protein 1	-	-
	IL1RAP	-0.056	16.078	-3.903	2.284E-04	0.030	-0.336	Down	interleukin 1 receptor accessory protein	Environmental Information Processing;	Signal transduction; Signaling molecules

										Organismal Systems	and interaction; Sensory system; Immune system
	SPARC	-0.377	15.424	-4.643	2.994E-05	0.020	2.364	Down	secreted protein acidic and cysteine rich	-	-
	SERPINB1	-0.444	15.420	-4.189	1.452E-04	0.034	0.939	Down	serpin family B member 1	-	-
	PRSS3	-0.192	17.217	-4.130	1.553E-04	0.034	0.834	Down	serine protease 3	Organismal Systems; Human Diseases; Environmental Information Processing	Digestive system; Infectious diseases; Signaling molecules and interaction
HDL	AFM	-0.034	22.766	-3.850	3.306E-04	0.043	0.111	Down	afamin	-	-
	**GPLD1**	-0.054	20.211	-3.835	3.466E-04	0.043	0.068	Down	glycosylphosphatidylinositol specific phospholipase D1	Metabolism	Global and overview maps; Glycan biosynthesis and metabolism
	**BCHE**	-0.044	20.141	-3.796	3.918E-04	0.043	-0.045	Down	butyrylcholinesterase	-	-
	**EFEMP1**	0.024	19.259	6.154	1.101E-07	7.321E-05	7.230	Up	EGF containing fibulin extracellular matrix protein 1	-	-
GGT	**IGFALS**	-0.034	20.471	-5.594	8.361E-07	2.780E-04	5.237	Down	insulin like growth factor binding protein acid labile subunit	Organismal Systems	Endocrine system
	**IGF2**	-0.020	20.257	-5.183	3.625E-06	8.035E-04	3.801	Down	insulin like growth factor 2	Environmental Information Processing; Human Diseases	Signal transduction; Cancers
	**CFD**	0.021	19.079	4.262	8.552E-05	0.014	0.731	Up	complement factor D	Human Diseases;	Infectious diseases;

	PRSS3	0.027	17.245	4.090	1.720E-04	0.023	0.130	Up	serine protease 3	Organismal Systems Organismal Systems; Human Diseases; Environmental Information Processing	Immune system Digestive system; Infectious diseases; Signaling molecules and interaction
	FBLN1	0.013	20.993	3.754	4.409E-04	0.042	-0.841	Up	fibulin 1	-	-
	**IGFBP3**	-0.031	20.465	-3.728	4.782E-04	0.042	-0.918	Down	insulin like growth factor binding protein 3	Human Diseases; Cellular Processes; Organismal Systems	Cancers; Cell growth and death; Endocrine system
	**CD14**	0.015	19.375	3.650	6.096E-04	0.042	-1.149	Up	CD14 molecule	Cellular Processes; Human Diseases; Organismal Systems; Environmental Information Processing	Transport and catabolism; Infectious diseases; Immune system; Cancers; Signal transduction
	**CD44**	0.019	18.584	3.638	6.320E-04	0.042	-1.184	Up	CD44 molecule (Indian blood group)	Human Diseases; Organismal Systems; Environmental Information Processing	Infectious diseases; Immune system; Cancers; Signaling molecules and interaction
GOT	PRSS3	0.059	17.245	7.133	5.779E-09	3.843E-06	10.338	Up	serine protease 3	Organismal Systems; Human Diseases; Environmental Information Processing	Digestive system; Infectious diseases; Digestive system; Signaling molecules and interaction
	**EFEMP1**	0.024	19.259	5.133	4.319E-06	0.001	3.789	Up	EGF containing fibulin	-	-

									extracellular matrix protein 1		
	**IGFALS**	-0.037	20.471	-5.071	5.369E-06	0.001	3.577	Down	insulin like growth factor binding protein acid labile subunit	Organismal Systems	Endocrine system
	**CD14**	0.020	19.375	4.167	1.171E-04	0.014	0.600	Up	CD14 molecule	Cellular Processes; Human Diseases; Organismal Systems; Environmental Information Processing	Transport and catabolism; Infectious diseases; Immune system; Cancers; Signal transduction
	**CD44**	0.026	18.584	4.156	1.212E-04	0.014	0.568	Up	CD44 molecule (Indian blood group)	Human Diseases; Organismal Systems; Environmental Information Processing	Infectious diseases; Immune system; Cancers; Signaling molecules and interaction
	**IGF2**	-0.020	20.257	-4.141	1.271E-04	0.014	0.521	Down	insulin like growth factor 2	Environmental Information Processing; Human Diseases	Signal transduction; Cancers
	**VCAM1**	0.036	16.235	3.982	2.133E-04	0.020	0.027	Up	vascular cell adhesion molecule 1	Environmental Information Processing; Human Diseases; Organismal Systems	Signaling molecules and interaction; Infectious diseases; Immune system; Signal transduction; Cardiovascular diseases; Endocrine

											and metabolic diseases
	**ITIH3**	0.021	21.414	3.824	3.530E-04	0.026	-0.454	Up	inter-alpha-trypsin inhibitor heavy chain 3	-	-
	KLKB1	-0.011	22.778	-3.802	3.787E-04	0.026	-0.520	Down	kallikrein B1	Organismal Systems	Immune system
	**CNDP1**	-0.030	19.823	-3.795	3.874E-04	0.026	-0.542	Down	carnosine dipeptidase 1	Metabolism	Global and overview maps; Amino acid metabolism; Metabolism of other amino acids
	**BCHE**	-0.021	20.150	-3.672	5.693E-04	0.034	-0.907	Down	butyrylcholinesterase	-	-
	PRSS3	-0.100	17.108	-7.079	1.697E-09	1.130E-06	11.066	Down	serine protease 3	Organismal Systems; Human Diseases; Environmental Information Processing	Digestive system; Infectious diseases; Signaling molecules and interaction
	IGLV9.49	-0.126	20.821	-6.706	6.092E-09	2.029E-06	9.781	Down	-	-	-
	ARHGDIB	-0.104	17.636	-4.731	1.400E-05	0.003	2.195	Down	Rho GDP dissociation inhibitor beta	Organismal Systems	Nervous system; Excretory system
DBIL	ABRACL	-0.098	15.541	-4.698	2.237E-05	0.004	1.797	Down	ABRA C-terminal like	-	-
	PLXDC2	-0.094	16.109	-4.139	1.089E-04	0.015	0.207	Down	plexin domain containing 2	-	-
	SERPINA4	-0.013	21.179	-4.033	1.436E-04	0.016	-0.076	Down	serpin family A member 4	-	-
	PGAM1	-0.098	15.589	-3.907	2.543E-04	0.024	-0.592	Down	phosphoglycerate mutase 1	Metabolism; Human Diseases; Organismal Systems	Global and overview maps; Carbohydrate metabolism; Cancers; Endocrine system; Amino acid

											metabolism
	SPARC	-0.090	15.262	-3.853	2.900E-04	0.024	-0.726	Down	secreted protein acidic and cysteine rich	-	-
	PGD	-0.096	15.123	-3.762	3.808E-04	0.024	-0.994	Down	phosphogluconate dehydrogenase	Metabolism	Global and overview maps; Carbohydrate metabolism; Metabolism of other amino acids
	CORO1A	-0.104	17.645	-3.758	3.994E-04	0.024	-1.029	Down	coronin 1A	Cellular Processes; Human Diseases	Transport and catabolism; Infectious diseases
	IGHV3.73	-0.016	21.142	-3.666	4.892E-04	0.027	-1.246	Down	-	-	-
	SERPINB1	-0.098	15.339	-3.650	5.710E-04	0.029	-1.367	Down	serpin family B member 1	-	-
	IGLV9.49	-0.101	20.842	-6.813	3.715E-09	2.474E-06	10.070	Down	-	-	-
	PRSS3	-0.075	17.107	-6.368	2.637E-08	8.780E-06	8.147	Down	serine protease 3	Organismal Systems; Human Diseases; Environmental Information Processing	Digestive system; Infectious diseases; Signaling molecules and interaction
	ABRACL	-0.083	15.541	-5.050	6.802E-06	0.002	2.762	Down	ABRA C-terminal like	-	-
TBIL	ARHGDIB	-0.079	17.636	-4.457	3.704E-05	0.006	1.042	Down	Rho GDP dissociation inhibitor beta	Organismal Systems	Nervous system; Excretory system
	SERPINA4	-0.011	21.182	-4.147	9.557E-05	0.013	0.100	Down	serpin family A member 4	-	-
	PLXDC2	-0.074	16.136	-4.033	1.533E-04	0.017	-0.329	Down	plexin domain containing 2	-	-
	PGAM1	-0.080	15.589	-3.993	1.920E-04	0.018	-0.531	Down	phosphoglycerate mutase 1	Metabolism; Human Diseases; Organismal Systems	Global and overview maps; Carbohydrate metabolism;

											Cancers; Endocrine system; Amino acid metabolism
	SERPINB1	-0.083	15.339	-3.913	2.456E-04	0.020	-0.771	Down	serpin family B member 1	-	-
	SPARC	-0.070	15.252	-3.723	4.366E-04	0.029	-1.325	Down	secreted protein acidic and cysteine rich	-	-
	IGHV3.73	-0.012	21.135	-3.614	5.741E-04	0.034	-1.615	Down	-	-	-
	CORO1A	-0.082	17.645	-3.622	6.161E-04	0.034	-1.647	Down	coronin 1A	Cellular Processes; Human Diseases	Transport and catabolism; Infectious diseases

Bold shows the hub proteins which correlated with both physiological index and age.

Supplemental Table S3. Hub proteins and top 20 keywords co-occurrence cluster analysis.

Rank	Hub Proteins	Count	Correlation
1	CD14	329	2247
2	CD44	267	1432
3	CFD	208	627
4	IGF2	174	1003
5	LBP	81	349
6	IGFBP-3	71	493
7	EFEMP1	29	151
8	AHSG	18	105
**Rank**	**Keyword**	**Count**	**Correlation**
1	inflammation	490	3311
2	disease	351	2245
3	age	368	2140
4	health	204	1374
5	aging	216	1288
6	obesity	167	1204
7	mortality	163	1075
8	monocytes	154	1019
9	exercise	145	1003
10	differentiatin	184	986
11	cytokines	137	908
12	atherosclerosis	136	903
13	growth	158	835
14	immune activation	106	830
15	c-reactive protein	112	804
16	physical-activity	101	764
17	infection	117	760
18	primary-care	112	755
19	microbial translocation	99	753
20	oxidative stress	119	629

Supplemental Figures

Supplemental Figure S1. Four clusters of plasma proteome trajectories.

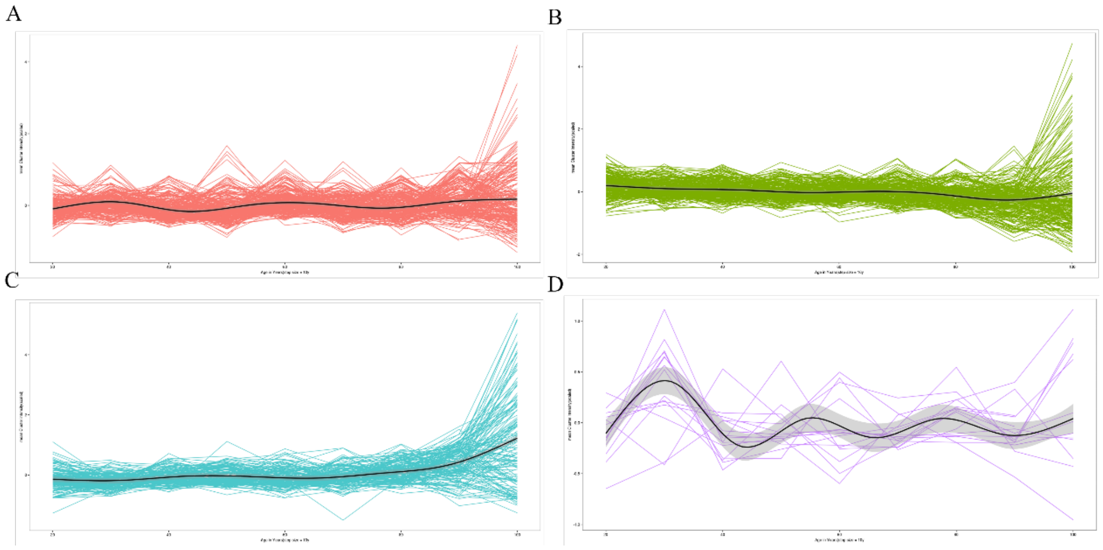

Supplemental Figure S2. The characteristics of physiological indices.

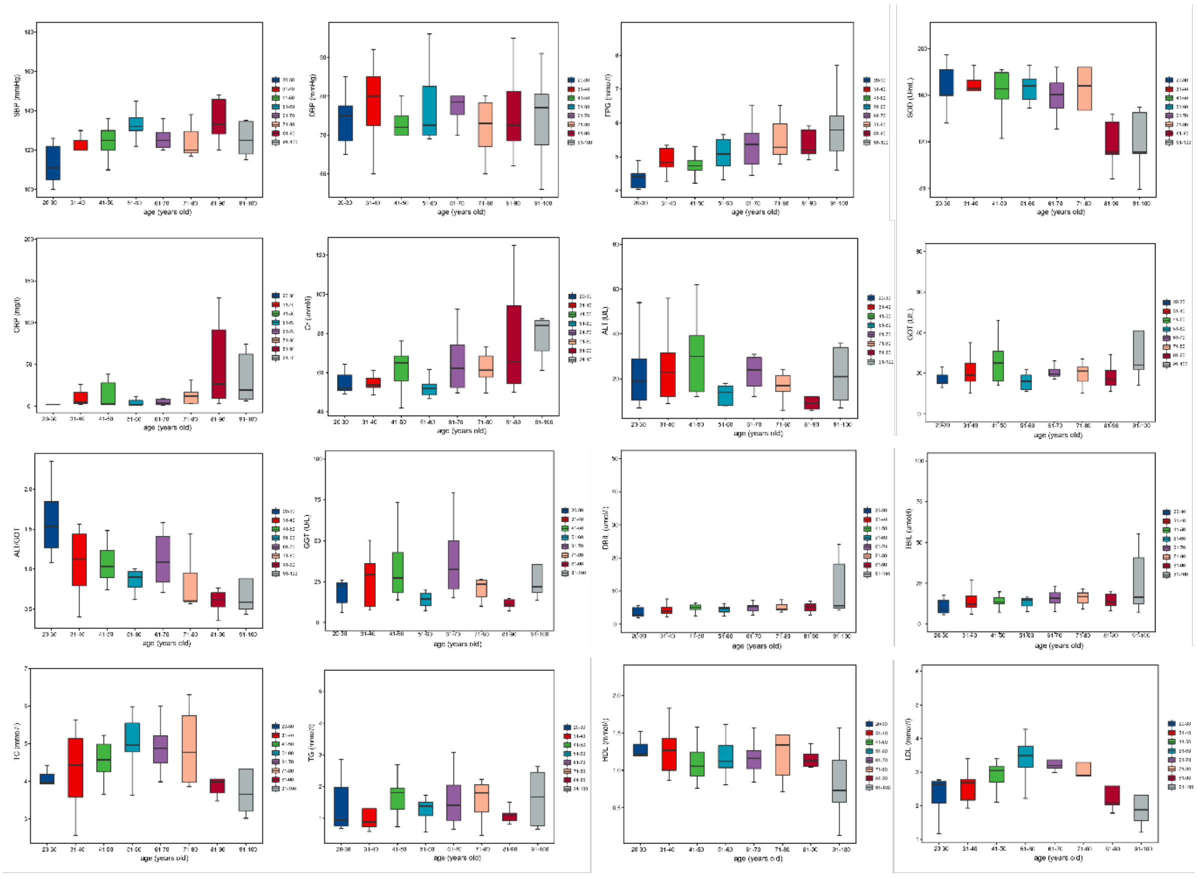

Supplement: Supplementary file 1 — Supplementary figures and tables. [file ijmsv22p1109s1.pdf]
